# Supplementary material for: Spatial distribution and determinants of improved shared sanitation facilities among households in Ethiopia: Using 2019 mini-Ethiopian Demographic and Health Survey
Source: PLoS One. 2025 Jan 13;20(1):e0315860. doi: 10.1371/journal.pone.0315860 (PMC11730421; doi:10.1371/journal.pone.0315860)
Supplement: Attachment — Submitted filename: PONE-D-24-20290_R1.pdf [file pone.0315860.s002.pdf]

PLOS ONE

Spatial distribution and determinants of improved shared sanitation facilities among households in Ethiopia: Using 2019 mini-Ethiopian Demographic and Health Survey

--Manuscript Draft--

**Manuscript Number:**	PONE-D-24-20290R1
**Article Type:**	Research Article
**Full Title:**	Spatial distribution and determinants of improved shared sanitation facilities among households in Ethiopia: Using 2019 mini-Ethiopian Demographic and Health Survey
**Short Title:**	Spatial distribution and determinants of improved shared sanitation facilities among households in Ethiopia
**Corresponding Author:**	Baye Amlak<br>Debre Markos University College of Health Science<br>Debre Markos, ETHIOPIA
**Keywords:**	Shared toilet, EDHS 2019, determinant, spatial, households, Ethiopia
**Abstract:**	ABSTRACT<br>Introduction: Limited or shared sanitation services are considered improved sanitation facilities, but they are shared between two or more households. Globally, 600 million people use shared toilet facilities. Although shared facilities are not classified as improved sanitation due to potential infection risks, inaccessibility, and safety concerns, this is a significant issue in developing countries like Ethiopia. Evidence on the distribution of shared sanitation services and their determinants in Ethiopia is limited. Therefore, this study aimed to assess the extent of shared toilet facilities and their determinants among households in Ethiopia.<br>Methods: The 2019 Ethiopian Demographic and Health Survey (EDHS) served as the basis for the cross-sectional secondary data analysis. The analysis included a total of 7,770 households from the weighted sample. STATA 14 software was used to clean, weigh, and analyze the data. To explore the distribution and determine the factors associated with shared toilet facilities in Ethiopia, both spatial and mixed-effect analyses were utilized. A p-value of less than 0.05 was used to display the relationships between the dependent and independent variables, employing adjusted odds ratios and 95% confidence intervals.<br>Results: The magnitude of improved shared sanitation facilities among households in Ethiopia, according to the EDHS 2019, was 10.5% (95% CI: 9.88, 11.24). The prevalence was highest in Addis Ababa at 70.2% and lowest in the Southern Nations, Nationalities, and Peoples' Region at 2.4%. Individual-level variables significantly associated with the use of improved shared toilet facilities included being a household head aged 55 years or older [AOR=0.48; 95% CI: 0.33, 0.71], having secondary education or higher [AOR=2.43; 95% CI: 1.80, 3.28], and belonging to middle or rich wealth status [middle: AOR=2.32; 95% CI: 1.35, 3.96; rich: AOR=6.23; 95% CI: 3.84, 10.11]. Community-level characteristics such as residing in urban areas [AOR=7.60; 95% CI: 3.47, 16.67], the metropolitan region [AOR=25.83; 95% CI: 10.1, 66.3], and periphery regions [AOR=5.01; 95% CI: 2.40, 10.48] were also associated with the use of shared toilet facilities.<br>Conclusion: The usage of improved shared toilet facilities among households in Ethiopia is relatively low. Significant factors related to the use of shared toilet facilities were being 55 years of age or older, possessing secondary or higher education, having a middle or rich wealth status, living in urban areas, and residing in metropolitan or peripheral regions. To improve access to and utilization of shared sanitation facilities, Ethiopian policy should emphasize user education and awareness.
**Order of Authors:**	Baye Tsegaye Amlak
	Daniel Gashaneh Belay
**Response to Reviewers:**	Date: September 1, 2024<br>Alison Parker<br>Academic Editor of Plos One Journal<br>Re: Spatial distribution and determinants of improved shared toilet facilities among households in Ethiopia: Using 2019 mini-Ethiopian Demographic and Health Survey

Powered by Editorial Manager® and ProduXion Manager® from Aries Systems Corporation

(Submission ID: PONE-D-24-20290)
Dear Editor,
We are grateful for the opportunity to revise our manuscript for further consideration for publication in Plos One Journal.
We have addressed the reviewer's comments and suggestions. Our point-by-point response describes all changes in the manuscript text. We have indicated the changes in track changes in the revised manuscript. We hope that you will find the revised manuscript acceptable for publication.
Yours sincerely,

Baye Tsegaye Amlak
Corresponding Author

A point-by-point response to the reviewer’s comment
We thank the editor and reviewer for their time and effort in reviewing our manuscript and for highlighting its importance. We have addressed the comments provided and revised the manuscript accordingly.
Reviewer 1
Overall: This paper addresses a critical issue of shared sanitation and its role in reaching the goal of safely managed sanitation by 2030. Understanding the extent of shared sanitation use in Ethiopia may support strategies for realising SDG6 and for understanding what the barriers to achieving this might be.
Response: Certainly, Thank you for your critical feedback and for recognizing the importance of this manuscript. We have addressed the comments provided and revised the manuscript accordingly.
Comment #1. I think that there is a useful paper in here, but there needs to be a bit more clarity about what is arising from the study, what is new in the study, and what is a confirmation of other studies on the issue of shared sanitation. It also needs to be clearer when the other literature is arising from a case study in a particular country, and so may not be generalisable (will clarify below in the line by line review when this comes up). I think that there are times that other studies are used to illustrate something that does NOT arise out of this study (for example on domestic violence) and this is confusing to the reader.
Response: Thank you for noting this. This study focused only on improved shared sanitation and its spatial distribution in Ethiopia. Providing shared sanitation services where improved sanitation services are limited could be a crucial first step in achieving the universal sanitation coverage aim outlined in the Sustainable Development Goals. We use only variables which have been associated with the previous studies on sanitation services.

Comment #2. The study could benefit from more clarity on the definitions used, particularly given the move from MDGs to SDGs and the greater focus on whether a service is safely managed rather than on the technologies used.
Response: Thank you for noting this. The definition of the outcome variable is clearly stated in the "Study Variables" section. We have revised it based on the DHS recode manual to read: "If a household uses any of the listed improved sanitation facilities and shares them with other households it is considered as improved shared sanitation services" (see page 7, lines 153-155).
Comment #3. One important clarification would be whether this study assumes that people who are not using SS are practising OD – is that correct? If so, that needs to be spelled out – that SS is the better option. Because at the moment we just read, those who use SS and those who do not, but without the clarity of what the options are of those who do not use SS. This could be my lack of statistical knowledge, but even so, it would be good to clarify in the text. To the same end, it would be useful to have a graphic of the overall breakdown of sanitation used in Ethiopia – from SMS, to SS, to OD.
Response: Thank you for noting this. We have corrected it; accordingly, the following sentences clearly shows the outcome and controls. The outcome of this study is improved shared sanitation facilities whereas the control group are all unimproved sanitation facilities which includes OD, and all unimproved sanitation services such as Pit latrines without slab/open pits, Bucket toilets, hanging toilet/latrines and others. We modified and revised the title and all sections of the manuscript accordingly. “Spatial distribution and determinants of improved shared sanitation facilities among households in Ethiopia: Using 2019 mini-Ethiopian Demographic and Health Survey”

Powered by Editorial Manager® and ProduXion Manager® from Aries Systems Corporation

The study population consisted of all houses that had unimproved sanitation services evaluated during the 2019 mini-EDHS survey (7,561). This includes, shared improved sanitation services (1,276), unimproved sanitation facilities (3,442), and open defecation (OD) (2,843). From the total 8,663, households included in the 2019 mini-EDHS survey, 1,102 households did have improved but not shared sanitation facilities and were excluded from the analysis. Ultimately, a sample of 7,561 (weighted 7,770) households was included in the analysis. (see Page 6 line no 146-151).

Comment #4. For the results/discussion/conclusion, you could provide recommendations that arise from this study – what does the data tell you that will help planners etc. to increase access to shared sanitation, or more specifically to move people from OD to shared sanitation?

Response: Thank you for noting this. We have added a recommendations section and incorporated the issues that need to be addressed by the concerned bodies and stakeholders. Revised according to the following “The government of Ethiopia and other stakeholders should provide economic support to low-income households to construct shared sanitation facilities. Additionally, policy should emphasize user awareness and practices through education to enhance access to and usage of improved shared sanitation facilities, particularly in rural residences and large central regions. Further qualitative studies are needed to explore the behavioural and socio-cultural factors that may prevent individuals from utilizing improved shared sanitation facilities” (see page 20, lines 385-391).

Abstract

Comment #5. Line 29 -33 This starts as a good argument for including shared toilets in statistics but lines 33-34 do not clarify how understanding the prevalence of shared sanitation (SS) further supports this argument.

Response: Thank you for highlighting this important point. We have addressed the identified gap and incorporated it to support the argument. Corrected as follows: “Evidences are limited on the distribution of shared sanitation services and its determinants in Ethiopia. Therefore, this study aimed to assess the magnitude of shared toilet facilities and their determinants among households in Ethiopia.” (see Abstract page 2, lines 30-31 And page 5 line number line number 118-119 in the introduction section).

Comment #6. Lines 35-50 – I am not a statistician so cannot comment

Response: Thank you for your honest feedback, we double-checked it.

Comment #7. Lines 51-54, It would be useful to have a few headline results – what age or education means for the prevalence of shared sanitation

Response: Thank you for your valuable comment. We have corrected it as follows: “Being 55 years or older, having secondary or higher education, having middle or rich wealth status, living in urban areas, and residing in metropolitan or peripheral regions were significantly associated with the usage of shared toilet facilities.” (see page 3, lines 51-53).

Introduction

Comment #8.

I recommend that the discussion on the move from MDGs to SDGs happens here, and what that means for the indicators etc., as the data is from the SDG era.

e.g. lines 70-78 – are these MDG definitions, or SDG definitions

Response: Thank you for your response The SDG definition states that “The Sustainable Development Goals target 6.2 calls for universal access to sanitation by 2030 and no child should die or get sick as a result of drinking contaminated drinking water, and/or being exposed to other people's excreta”

Comment #9. Line 79 – check the numbers, something is not right – an increase of 3-6 million is not worth mentioning if the total using SS is 600 million. Line 80-82 – check the numbers 0.96 is not 18% of the population of SSA

Response: Revised accordingly as “Whereas in Sub-Saharan African countries households who used shared toilet facilities climbed from 0.64 million to 0.96 million, which was 0.08% of all households” (see page 2, lines 93-94).

Comment #10. Line86 – is a word missing after ‘easy’

Response: We thank you. It was a grammatical and typing error. Modification has been made throughout the document.

Comment #11. Lines 103-104 – what is the source of this statement (it is not SDG 6.2 as suggested by the reference)

Powered by Editorial Manager® and ProduXion Manager® from Aries Systems Corporation

Response: We thank you and revised the source.
https://iris.who.int/bitstream/handle/10665/258617/9789241512893-eng.pdf?sequence=1
Comment #12: Line 110 – they may be superior to OD – but what are the criteria?
Response: Thank you for raising this point: improved shared sanitation facilities are superior to open defecation for several reasons:
 They reduce the incidence of open defecation, lowering exposure to harmful pathogens and disease spread.
 They offer more privacy and dignity than open defecation, which occurs in public spaces.
 They generally cause less environmental pollution compared to open defecation, which contaminates soil and water sources.
 They provide a more structured and accessible option, especially in urban or densely populated areas.
Comment #12: Lines 115-117 – are you drawing a link between using, or not using SS and child mortality? Are you drawing that from your data?
Response: Thank you for your interesting question, it is not from our data. It is based on the reference since it is an introduction, we want to show its burden. However, we revised the child mortality section.
Comment #13: Lines 118-119 – what is the relevance for planners – so that they can target particular households?
Response: Thank you for your interesting question. Knowing the magnitude of improved shared toilet facilities and their contributing factors in Ethiopia helps program planners allocate resources more effectively, design targeted interventions, and develop relevant policies. This understanding enables better planning and decision-making, leading to improved sanitation outcomes, enhanced public health, and more efficient use of resources by addressing the specific needs and challenges faced by different communities (see page 5, lines 121-123).
Comment #14: Lines 138-143 – I am confused by these numbers – there are 8663 households across 645 EAs? That doesn't make 20-30 households per EA – please clarify here.
Response: Thank you very much for showing the mistake we made; Now we revised accordingly, as the following "In the first stage, a total of 305 EAs (93 in urban areas and 212 in rural areas) were selected with probability proportional to EA size. The second stage was the careful selection of, on average, 25–30 homes (based on the 2019 EPHC frame)" (see page 6, lines 141-143).Though the revised sample size is 7,561, in the 2019 mini-EDHS 8663 households were included. Then 8,863/305=29 households on average.
Comment #15. I regret that the rest of the methods section doesn't mean anything to me!
Response: No worries; thank you very much for focusing on your experts.

Results
Comment #16. Line 212 – can you define what a male head of household denotes and what a female head of household denotes? Is a head of household only female if there is no adult male in the house? Or how is it defined?
Response: Thank you for seeking clarification. Yes, as you mentioned, the sociodemographic variables of the household data/record (HR) in DHS are collected for the head of households only. Then in the Ethiopian context, mostly if both male (father) and female (mother) are present in the household, the male is considered the household head. However, if the female is leading the household alone, she is recognized as the household head. (See Table 2)
Comment #17: Line 215 I think here you could summarise up front that an increase in age, and decrease in education, wealth etc. leads to a household being more likely to use SS. Then you can go into the detail
Response: Thank you for this important comment. Revised in the manuscript as the following: "Based on the results from Model III, there was a positive association between improved shared sanitation facilities and several factors such as individuals with higher educational attainment and from greater household wealth, those living in urban areas and metropolitan regions". (see page 13, lines 244-246).

Comment #18: Lines 235 – 239 Where is the risk of DV data coming from? And is SS a DV risk or a GBV risk? What is the correlation? does this suggest a correlation? A causation? Or just two random connected indicators of increased vulnerability?

	Response: Thank you for your comments, and we apologize for any confusion. We are not entirely sure about the abbreviations "DV" and "GBV" you used. However, if "DV" refers to the dependent variable, it implies that improved shared sanitation facilities in our context. The risk associated with the dependent variable is influenced by the cluster in which individuals reside. Since we used Enumeration Area (EA) or cluster as a random variable, the variations observed in the null model (e.g., ICC, MOR) were not due to random chance but rather to the variability of the random variable, which was the cluster or enumeration area. This indicates there was a correlation or association between the random variable (cluster) and the dependent variable. This is accounted for by our multilevel analysis.<br>Comment #19: Line 244 – 247 – write this as clearly as possible also for a non-statistician audience – as I understand it, this is your main finding.<br>Response: Thank you for your suggestion: Modifications and clarification have been made in the manuscript as follows: "Based on the results from Model III, there was a positive association between improved shared sanitation facilities and several factors such as individuals with higher educational attainment and from greater household wealth, those living in urban areas and metropolitan regions". (see page 13, lines 244-246).<br>Comment #20: From line 306 onwards I cannot comment! Sorry!<br>Response: No worries; thank you very much for focusing on your experts.<br>Discussion<br>Comment #21: Again – I think it needs to be clarified what the non-SS households are using, to be clear that it is worse than SS.<br>Response: Thank you for your suggestion: We have corrected it; accordingly, the following sentences show the outcome and controls. The outcome of this study is improved shared sanitation facilities whereas the control group are all unimproved sanitation facilities which includes OD, and all unimproved sanitation services such as Pit latrines without slab/open pits, Bucket toilets, hanging toilet/latrines and others. (see table 1).<br>Comment #22: Line 329 - this is not a strong reference, as it is a webpage without links to the claims it references.<br>Response: Thank you for your suggestion: revised it accordingly.<br>Comment #23: Lines 331-332 Did you get this from the data in the study? Or from somewhere else (if so reference) Also add "Studies in Zambia and Ethiopia show….." I think that the context is critical.<br>Response: We have got it from previous studies, and we cited references accordingly as the following. "Moreover, a higher level of education enhances awareness and fosters positive attitudes towards choosing relatively more upgraded sanitation facilities [36]. Specifically, educated women are more likely to prefer safe and sanitary facilities that offer privacy and maintain good quality during their menstrual cycle [37]. Moreover, well-educated households with higher incomes had better access to upgraded sanitation facilities [38]. " (see page 18, lines 330-335).<br>Comment #24: Line 334 – is this an assumption? |Or does education lead to households using SS (which is what your data says, I think).<br>Response: Thank you for your valuable comments. Revised accordingly and removed the OD from the revised manuscript. Our data says household head education can lead to the use of Improved shared sanitation services and it is already expressed in the same paragraph.<br>Comment #25: Line 341 – the reference you use for the tragedy of the commons is rather old-fashioned, and the tragedy of the commons itself may not be relevant to sanitation usage. More work has been done on this more recently, e.g. by Ostrom, or by McGranahan.<br>Line 344 – 346 – is this speculation or from the data?<br>Line 346-348 it would be better to say "A study in Kenya shows…." Because otherwise it can be taken as a general truth and it may not be.<br>Response: Thank you for your valuable comments. We've updated and corrected the manuscript following the re-analysis. The number of family members in the household was found to be non-significant, and this discussion has been removed from the manuscript.<br>Comment #26: Line 347 – is 'consistent income' one of the indicators in the data, it's the first time I see the qualifier 'consistent'?<br>Response: Thank you for your comments. We discuss this issue for the association of rural residence and low improved sanitation facility usage from previous studies as the following

Powered by Editorial Manager® and ProduXion Manager® from Aries Systems Corporation

“On the other side, sanitation is an investment and peri-urban inhabitants made the effort to get improved toilet facilities because they had relatively consistent income to support their planning.” (see page 18, lines 352-354).
Comment #27: Lines 358 – 361 – this seems to be a circular argument – rewrite for clarity.
Response: Thank you for the comment. The information has been modified accordingly.
Comment #28: Line 361 – does ‘governments’ here refer to local governments in Ethiopia or other national governments?
Response: Both Ethiopian and other national governments do not prioritize rural sanitation specifically in their national agenda.
Comment #29: Line 365 – blanket statement not supported by the reference as far as I can see
Response: Thank you. Just reference is cited.

Conclusion
Comment #30: The conclusion could usefully contain some recommendations arising from the study – how can planners/government used this data analysis to make changes to how they promote SS? What are the lessons that can be acted upon?
Response: Thank you for your comments; we revised the recommendation in the revised manuscript as the following “The government of Ethiopia and other stakeholders should provide economic support to low-income households and rural residents to construct improved shared sanitation facilities. Additionally, policy should emphasize user awareness and practices through financial and education literacy to enhance access to and usage of improved shared sanitation facilities, particularly in rural residences and large central regions. Further qualitative studies are needed to explore the behavioural and socio-cultural factors that may prevent individuals from utilizing improved shared sanitation facilities (see page 20, lines 385-391).

Comment #31: What still remains to be researched and analysed. What is new from this study, and what confirms previous studies?
Response: Thank you for the interesting question. We reviewed various pieces of literature on the concept of shared sanitation and included them in the introduction. However, we found that no studies have specifically addressed the distribution of shared toilet facilities and their determinant factors in Ethiopia. Therefore, this study aims to address this gap. The new findings of this study are: we explored the spatial distribution and identified the determinants of improved shared sanitation facilities among households in Ethiopia. We suggested that further qualitative studies are needed to explore the behavioural and socio-cultural factors that may prevent individuals from utilizing improved shared sanitation facilities. (See page 20, lines 390-391).

Comment #33: Also – be careful what assumptions are embedded in the analysis – for example, if women care so much about sanitation, why do households with a male head of household have increased access to SS?
Response: Thank you for the comment. However, in our analysis, the gender of head of the household has no association with improved shared sanitation facility usage.
Comment #22: Another still to be resolved question, critical for acceptance of SS as safely managed sanitation is what types of shared toilet facility people are using, and whether they are properly maintained? I believe that the main issue with shared sanitation is that it is difficult to judge if they are indeed improved, or safely managed or barely better than OD.
Response: Thank you for the comment: The analysis included improved shared toilet facilities as an outcome variable. We revised accordingly starting from the title of the manuscript. “Spatial distribution and determinants of improved shared sanitation facilities among households in Ethiopia: Using 2019 mini-Ethiopian Demographic and Health Survey”
Reviewer #2:
Comment #1: Summary and overall impression.
The paper addresses important issue of shared sanitation facilities and puts it in the individual household and community perspective. As it uses well recognised and reliable EDHS survey data, the analysis should yield dependable results that could support policy decision makers and other stakeholders when developing interventions at local and country level. It should also provide reference points for researchers and

Powered by Editorial Manager® and ProduXion Manager® from Aries Systems Corporation

sanitation experts alike.
From a reader point of view there is a confusion related to the definition of shared sanitation and improved sanitation. I find it difficult to see how shared sanitation is assessed in the context of the type of primary toilet type available to the household. In terms of proportions, methodology and modelling sections are broad and detailed but the outcome of the analysis that is supposed to address research questions is incomplete.
Although the discussion section provides many valid points, the paper in my opinion requires major revisions to communicate its points clearly and develop more substantial recommendations that should be developed in the closing (conclusions) section.
Grammar, spelling mistakes and clarity of writing style must be addressed as well.
Evidence and examples
Response: Thank you for recognizing the importance of this manuscript and finally your overall critical feedback.
•The definition of improved shared sanitation is critically revised in the revised manuscript Table 1. The outcome of this study is improved but shared sanitation facilities. If a household uses any of the listed -improved sanitation facilities but shares them with other households, it is considered as improved shared sanitation facilities. The control group are all unimproved sanitation facilities which include OD, and sanitation services such as Pit latrines without slab/open pits, Bucket toilets, hanging toilets/latrines and others. We revised accordingly starting from the title of the manuscript
•Discussion and conclusion: The conclusion of the manuscript is revised based on the research questions and or objects. We have 3 research questions. 1. What was the magnitude of improved shared sanitation facilities in Ethiopia? 2. What do the spatial distributions of improved shared sanitation facilities in Ethiopia look like? 3. What are the associated factors with improved shared sanitation facilities?
•Therefore, we revised all the results and discussions accordingly as the following "The prevalence of improved shared sanitation services usage among households in Ethiopia is relatively low. Age, educational attainment of the household head, wealth status, residence, and region were found to be significantly associated factors with improved shared sanitation facilities in Ethiopia. Moreover, there was a non-random distribution of improved shared sanitation services in Ethiopia mainly found in Addis Ababa, Dirie Dawa". (see page 20, lines 381-385).
•Grammar and spelling mistakes: We have revised all the sections of the manuscript for grammar and spelling errors.

Major issues
Comment #2: The introduction that is to include an overview of previous research is narrow and lacks currency. It should have a clearer structure and include more recent research-based evidence. Indeed, the most recent reference cited is from 2022 (only one position).
I recommend Shared sanitation in informal settlements: A systematic review and meta-analysis of prevalence, preferences, and quality.
https://doi.org/10.1016/j.ijheh.2024.114392. to provide broader perspective for this paper in the context of previously published research.
Response: Thank you for your valuable feedback and for providing the important reference article. We have incorporated and cited this article in the revised manuscript and have updated the introduction to reflect the latest literatures (See page 4 line No 85-88 and page 17 line 310 in discussion part).

Comment #3: Definition of shared sanitation is unclear – contradicting statements 78 vs 75.
Response: Thank you for your critical comments. The definition of improved shared sanitation is critically revised in the revised manuscript Table 1. The outcome of this study is improved but shared sanitation facilities. If a household uses any of the listed -improved sanitation facilities but shares them with other households (literally unimproved), it is considered as improved shared sanitation facilities.(Improved sanitation service, if shared=unimproved).For more evidence (see the introduction part page 5 line no 82-84).The control group are all other unimproved sanitation facilities which include OD, and sanitation services such as Pit latrines without slab/open pits, Bucket toilets, hanging toilets/latrines and others. We revised accordingly starting from the title of the manuscript.

Powered by Editorial Manager® and ProduXion Manager® from Aries Systems Corporation

	Statements 78 vs 75 are not contradicted. If improved sanitation facilities are shared between two or more households, it is considered unimproved. Therefore, the term “improved shared sanitation” is one of unimproved sanitation services. The difference from other unimproved services such as OD is, that it is the acceptable and recommended service for refugees, slum areas and people with low socioeconomic status.<br>Comment #4: Research question 2 (118, 119) is unclear – contributing factors (contributing to what?).<br>Response: Thank you for your critical comments. The study is a cross-sectional study, we can assess only the association between Dependent Variable and independent variables. Therefore, we revised it accordingly as the following “What factors are associated with improved shared sanitation facilities?”<br>Comment #5:<br>The results section needs to be re-written to address gaps (e.g. explanation of how cluster with high risk of domestic violence was identified/taken from original data set?) and improve readability by providing more detailed interpretations (see e.g. 257, 258).<br>Response: Thank you for your comments. However, we had no domestic violence factor or outcome variables. Apologise I couldn't get your comments. For any variable generation from the original data set you can refer table Table 2: a list of the study's variables along with an explanation of each measurement<br>Minor issues<br>Comment #6: More recent population figures are available for Ethiopia now; 2023 as opposed to cited value for 2021 (131).<br>Response: Thank you for your comments: Revised based on the 2024, worldometere report. With 1.1 million square kilometres under its belt and an expected 132,059,767 inhabitants in 2024, Ethiopia is the second most populated country in Africa next to Nigeria<br>Comment #5: Table 1 (149) does not present the logic of questions well.<br>Response: Thank you for your comments, we revised Table 1 as the following:<br>Table 1: Questions to measure improved but shared/ limited sanitation service.<br>Outcome variable (Shared/ limited sanitation service)Control group/comparators<br>Did the toilet have flush/pour flush to the piped sewer system?Unimproved sanitation services such as<br>oPit latrine without slab/open pit<br>oBucket toilet<br>oHanging toilet/latrine<br>oOther<br>Open defecation: no facility/bush/field<br>Did the toilet have flush/pour flush to the septic tank?<br>Did the toilet have a flush to somewhere else?<br>Did the toilet have flush/pour flush to the pit latrine?<br>Did the toilet have a flush, don't know where?<br>Was the toilet Ventilated Improved Pit (VIP) latrine?<br>Was the toilet pit latrine with a slab?<br>Was the toilet composting toilet?<br>If a household uses any of the listed above-improved sanitation facilities but shares them with other households.<br><br>Miscellaneous comments<br>Comment #5: Formatting of references requires attention – there are issues with the numbers and completeness of the records.<br>Response: Thank you so much, we revised the reference format and numbering accordingly.
**Additional Information:**	
**Question**	**Response**
**Financial Disclosure**<br><br>Enter a financial disclosure statement that describes the sources of funding for the work included in this submission. Review the submission guidelines for detailed	The author(s) received no specific funding for this work.

requirements. View published research articles from *PLOS ONE* for specific examples.<br><br>This statement is required for submission and **will appear in the published article** if the submission is accepted. Please make sure it is accurate.<br><br>**Funded studies**<br>Enter a statement with the following details:<br>• Initials of the authors who received each award<br>• Grant numbers awarded to each author<br>• The full name of each funder<br>• URL of each funder website<br>• Did the sponsors or funders play any role in the study design, data collection and analysis, decision to publish, or preparation of the manuscript?<br><br>Did you receive funding for this work?	
**Competing Interests**<br><br>Use the instructions below to enter a competing interest statement for this submission. On behalf of all authors, disclose any competing interests that could be perceived to bias this work—acknowledging all financial support and any other relevant financial or non-financial competing interests.<br><br>This statement is required for submission and **will appear in the published article** if the submission is accepted. Please make sure it is accurate and that any funding sources listed in your Funding Information later in the submission form are also declared in your Financial Disclosure statement.<br><br>View published research articles from *PLOS ONE* for specific examples.	The authors declare that they have no competing interests.

Powered by Editorial Manager® and ProduXion Manager® from Aries Systems Corporation

**NO authors have competing interests**<br><br>Enter: *The authors have declared that no competing interests exist.*<br><br>**Authors with competing interests**<br><br>Enter competing interest details beginning with this statement:<br><br>*I have read the journal's policy and the authors of this manuscript have the following competing interests: [insert competing interests here]*<br><br>* typeset	
**Ethics Statement**<br><br>Enter an ethics statement for this submission. This statement is required if the study involved:<br><br>• Human participants<br>• Human specimens or tissue<br>• Vertebrate animals or cephalopods<br>• Vertebrate embryos or tissues<br>• Field research<br><br>Write "N/A" if the submission does not require an ethics statement.<br><br>General guidance is provided below. Consult the submission guidelines for detailed instructions. **Make sure that all information entered here is included in the Methods section of the manuscript.**	N/A

**Format for specific study types**<br><br>**Human Subject Research (involving human participants and/or tissue)**<br>• Give the name of the institutional review board or ethics committee that approved the study<br>• Include the approval number and/or a statement indicating approval of this research<br>• Indicate the form of consent obtained (written/oral) or the reason that consent was not obtained (e.g. the data were analyzed anonymously)<br><br>**Animal Research (involving vertebrate animals, embryos or tissues)**<br>• Provide the name of the Institutional Animal Care and Use Committee (IACUC) or other relevant ethics board that reviewed the study protocol, and indicate whether they approved this research or granted a formal waiver of ethical approval<br>• Include an approval number if one was obtained<br>• If the study involved *non-human primates*, add *additional details* about animal welfare and steps taken to ameliorate suffering<br>• If anesthesia, euthanasia, or any kind of animal sacrifice is part of the study, include briefly which substances and/or methods were applied<br><br>**Field Research**<br><br>Include the following details if this study involves the collection of plant, animal, or other materials from a natural setting:<br>• Field permit number<br>• Name of the institution or relevant body that granted permission	
**Data Availability**<br><br>Authors are required to make all data underlying the findings described fully available, without restriction, and from the time of publication. PLOS allows rare exceptions to address legal and ethical concerns. See the PLOS Data Policy and FAQ for detailed information.	Yes - all data are fully available without restriction

A Data Availability Statement describing where the data can be found is required at submission. Your answers to this question constitute the Data Availability Statement and **will be published in the article**, if accepted.<br><br>**Important:** Stating 'data available on request from the author' is not sufficient. If your data are only available upon request, select 'No' for the first question and explain your exceptional situation in the text box.<br><br>Do the authors confirm that all data underlying the findings described in their manuscript are fully available without restriction?	
**Describe where the data may be found in full sentences. If you are copying our sample text, replace any instances of XXX with the appropriate details.**<br><br>• If the data are **held or will be held in a public repository**, include URLs, accession numbers or DOIs. If this information will only be available after acceptance, indicate this by ticking the box below. For example: *All XXX files are available from the XXX database (accession number(s) XXX, XXX.*).<br>• If the data are all contained **within the manuscript and/or Supporting Information files**, enter the following: *All relevant data are within the manuscript and its Supporting Information files.*<br>• If neither of these applies but you are able to provide **details of access elsewhere**, with or without limitations, please do so. For example:<br><br>*Data cannot be shared publicly because of [XXX]. Data are available from the XXX Institutional Data Access / Ethics Committee (contact via XXX) for researchers who meet the criteria for access to confidential data.*<br><br>*The data underlying the results presented in the study are available from (include the name of the third party*	Data were accessed after registration, submission of the request, and permission granted via the link https://dhsprogram.com/data/new-user-registration.cfm. The shapefile data was created by GADM, found here: https://gadm.org/index.html , and was created under this license.

and contact information or URL).<br>• This text is appropriate if the data are owned by a third party and authors do not have permission to share the data.<br><br>* typeset	
Additional data availability information:	

Manuscript

Click here to access/download;Manuscript;Manuscript shared toilet facilities revised _DB.docx

Spatial distribution and determinants of improved shared sanitation facilities among households in Ethiopia: Using 2019 mini-Ethiopian Demographic and Health Survey

Baye Tsegaye Amelak[1], Daniel Gashaneh Belay [2, 3],

[1]Department of Nursing, College of Medicine and Health Sciences, Debre Markos University, Debre Markos, Ethiopia, Email: bayetsegaye07@gmail.com

[2]Department of Human Anatomy, School of Medicine, College of Medicine and Health Sciences, University of Gondar, Gondar, Ethiopia. Email, danielgashaneh28@gmail.com

[3]Department of Epidemiology and Biostatistics, Institute of Public Health, College of Medicine and Health Sciences, University of Gondar, Gondar, Ethiopia

*Corresponding author

Baye Tsegaye Amlak (Department of Nursing, College of Health Sciences, Debre Markos University, Debre Markos, Ethiopia. Email: bayetsegaye07@gmail.com

ABSTRACT

Introduction: Limited or shared sanitation services are considered improved sanitation facilities, but they are shared between two or more households. Globally, 600 million people use shared toilet facilities. Although shared facilities are not classified as improved sanitation due to potential infection risks, inaccessibility, and safety concerns, this is a significant issue in developing countries like Ethiopia. Evidence on the distribution of shared sanitation services and their determinants in Ethiopia is limited. Therefore, this study aimed to assess the extent of shared toilet facilities and their determinants among households in Ethiopia.

Methods: The 2019 Ethiopian Demographic and Health Survey (EDHS) served as the basis for the cross-sectional secondary data analysis. The analysis included a total of 7,770 households from the weighted sample. STATA 14 software was used to clean, weigh, and analyze the data. To explore the distribution and determine the factors associated with shared toilet facilities in Ethiopia, both spatial and mixed-effect analyses were utilized. A p-value of less than 0.05 was used to display the relationships between the dependent and independent variables, employing adjusted odds ratios and 95% confidence intervals.

Results: The magnitude of improved shared sanitation facilities among households in Ethiopia, according to the EDHS 2019, was 10.5% (95% CI: 9.88, 11.24). The prevalence was highest in Addis Ababa at 70.2% and lowest in the Southern Nations, Nationalities, and Peoples' Region at 2.4%. Individual-level variables significantly associated with the use of improved shared toilet facilities included being a household head aged 55 years or older [AOR=0.48; 95% CI: 0.33, 0.71], having secondary education or higher [AOR=2.43; 95% CI: 1.80, 3.28], and belonging to middle or rich wealth status [middle: AOR=2.32; 95% CI: 1.35, 3.96; rich: AOR=6.23; 95% CI: 3.84, 10.11]. Community-level characteristics such as residing in urban areas [AOR=7.60; 95% CI: 3.47, 16.67], the metropolitan region [AOR=25.83; 95% CI: 10.1, 66.3], and periphery regions [AOR=5.01; 95% CI: 2.40, 10.48] were also associated with the use of shared toilet facilities.

Conclusion: The usage of improved shared toilet facilities among households in Ethiopia is relatively low. Significant factors related to the use of shared toilet facilities were being 55 years of age or older, possessing secondary or higher education, having a middle or rich wealth status, living in urban areas, and residing in metropolitan or peripheral regions. To improve access to and utilization of shared sanitation facilities, Ethiopian policy should emphasize user education and awareness.

Keywords: Shared toilet, EDHS 2019, determinant, spatial, households, Ethiopia

Introduction

The World Health Organization (WHO) and the United Nations Joint Monitoring Programme (JMP) define limited sanitation services as improved sanitation facilities that are shared between two or more households [1, 2]. Globally, 2.3 billion people lack access to improved sanitation facilities [2]. From these, 600 million people use shared toilet facilities [2].

Shared sanitation is the o[illegible]ption available to millions of people living in densely packed urban areas, especially in informal settlements. This is preferable to open defecation, which has far more severe negative impacts on health, safety, and dignity [1]. Furt[illegible]ore, while shared toilets are designed to be an improved type of sanitation facility, they are considered unimproved because they are shared among multiple users [3].

A meta-analysis of global studies on shared sanitation in informal settlements estimated that the overall prevalence of shared sanitation was 67%. Users' preferences for using shared facilities were influenced by factors such as cleanliness, affordability, safety, privacy, structural quality, and accessibility[4]. However, informal settlements are widespread and often characterized by substandard housing, poverty, and a lack of basic sanitation facilities. This is especially true in developing nations like Ethiopia, where conflict and internal displacement are prevalent [5]. Therefore, sharing toilets provides residents who lack private toilets in their homes with access to sanitation facilities [5].

In Sub-Saharan African countries, the number of households using shared toilet facilities increased from 0.64 million to 0.96 million, representing 0.08% of all households [2, 6]. Similarly, in Ethiopia, the use of shared toilet facilities rose from 4% to 7% between 2000 and 2015 [7]. Shared facilities are not regarded as an improvement in sanitation because they frequently encounter maintenance issues. Furthermore, their accessibility can lead to the spread of infections due to poor hygiene, limited accessibility, and unsafe conditions [8]. These are frequently not well kept, creating unclean conditions that discourage frequent use [1]. Global research has shown a

connection between the use of shared toilets and adverse health outcomes such as helminth infections, diarrhea, enteric fevers, and fecal-oral diseases [9]. It aids the transmission of microorganisms that cause diarrheal diseases [10], with children being the most vulnerable [11]. Diarrheal disease is the second leading cause of death for children under five worldwide, accounting for 760,000 deaths and 1.7 million morbidities annually [12]. In Africa, diarrhea is one of the main causes of death in under-five children [13], It causes the deaths of half a million children under the age of five annually in Ethiopia alone [14]. Moreover, using shared toilets puts hundreds of millions of women and children at greater risk of sexual exploitation and a lack of privacy during their menstrual cycles [15] [5]. Poor sanitation is also associated with infections and eye diseases, such as trachoma[5].

Sustainable Development Goal (SDG) target 6.2 aims to ensure that by 2030, all ch[illegible]n will have access to universal sanitation facilities [8] and that no child should suffer from disease or die due to contaminated water or contact with human waste [16]. Therefore, providing shared sanitation services could be a crucial initial step toward achieving the universal sanitation coverage goal set out in the Sustainable Development Goals [8]. Since 1995, Ethiopia has prioritized its sanitation program, following the inclusion of public health in the country's National Constitution. Subsequently, in 2005 and 2006, the Ministry of Health developed the National Hygiene and Sanitation Strategy and the National Hygiene and On-Site Sanitation Protocol, respectively[17, 18]. However, the magnitude and contributing factors to using shared toilet facilities in Ethiopia are little known [19]. Therefore, this study aims to address the following research questions: What is the magnitude and spatial distribution of improved shared sanitation facilities in Ethiopia? What factors are associated with improved shared sanitation facilities? Answering these questions will provide valuable insights for policymakers and program planners, helping them allocate resources more effectively, design targeted interventions, and develop relevant policies.

Methodology

Study design, setting, and data source

This study utilized data from the recent Ethiopian Demographic and Health Survey (EDHS 2019), which was collected using a community-based cross-sectional study design. Since 1984, the DHS has gathered a broad range of objective and self-reported data across more than 99 countries. Key advantages of the DHS include high response rates, national coverage, rigorous interviewer training, standardized data collection procedures across countries, and consistent content over time [20].

With 1.1 million square kilometers under its belt and an expected 132,059,767 inhabitants in 2024, Ethiopia is the second most populated country in Africa next to Nigeria [21]. Ethiopia has two city administrations (Addis Ababa and Dire Dawa) and nine regions (Tigray, Afar, Amhara, Oromia, Benishangul Gumuz, Somalia, South Nation Nationalities and Peoples of Ethiopia (SNNP), Gambelia, and Harari) with a federal decentralized administrative structure. To ensure survey precision was comparable across regions, the sample allocation was carried out through equal distribution, with 35 enumeration areas (EAs) selected from each of the three larger regions: Amhara, Oromia, and the Southern Nations, Nationalities, and Peoples' Region (SNNPR). Additionally, 25 EAs were selected from each of eight other regions. In the first stage, a total of 305 EAs (93 in urban areas and 212 in rural areas) were chosen with probability proportional to EA size. In the second stage, an average of 25–30 households were carefully selected from each EA based on the 2019 Ethiopian Population and Housing Census (EPHC) frame[22].

Study population

The study population consisted of all houses that had unimproved sanitation services evaluated during the 2019 mini EDHS survey (7,561). This includes, shared improved sanitation services (1,276), unimproved sanitation facilities (3,442), and open defecation (OD) (2,843).

Out of the 8,663 households included in the 2019 mini EDHS survey, 1,102 households had improved but not shared sanitation facilities and were excluded from the analysis. Ultimately, a sample of 7,561 households (weighted to 7,770) was included in the analysis.

Study variables

The outcome variable of the study was shared/ limited sanitation service which means households with either one type of improved sanitation facility but shared with other households [23] **(Table 1).**

Table 1: Questions to measure improved but shared/ limited sanitation service.

**Outcome variable (Shared/ limited sanitation service)**	**Control group/comparators**
Did the toilet have flush/pour flush to the piped sewer system?	✓ Unimproved sanitation services such as<br>○ Pit latrine without slab/open pit<br>○ Bucket toilet<br>○ Hanging toilet/latrine<br>○ Other<br>✓ Open defecation: no facility/bush/field
Did the toilet have flush/pour flush to the septic tank?	
Did the toilet have a flush to somewhere else?	
Did the toilet have flush/pour flush to the pit latrine?	
Did the toilet have a flush, *don't know where.*	
Was the toilet Ventilated Improved Pit (VIP) latrine?	
Was the toilet pit latrine with a slab?	
Was the toilet composting toilet?	
If a household uses any of the listed above-improved sanitation facilities but shares them with other households.	

The study's predictor variables were categorized into individual-level variables, which included the age, sex, and educational attainment of household heads, as well as factors such as the household wealth index, family size, and household size. Additionally, community-level characteristics such as residence location, region, and community poverty were examined. After assessing the normal distribution of the aggregated community components using a histogram and

the Shapiro-Wilk test, the data were recorded using the appropriate measures of central tendency **(Table 2).**

Table 2: ***A list of the study's variables along with an explanation of each measurement***

Level	Variables	Measurements
Individual level variables	Age	The age of the participants was categorized as 15-24, 25-40, 41-54, >55
	Sex	Sex of the household head as male and female
	Education level	Educational attainment is categorized as uneducated, primary, secondary, and above
	Family size	Categorized as 1-3, 4-6, and 7 and above.
	Wealth index	A wealth index categorized as poorest, intermediate, richest, and wealthiest in the DHS data collection was developed using principal components analysis and included in the datasets. We classified it into three categories for this study: middle class, rich (including wealthier), and poor (including poorer and poorest).
Community level variables	Residency	Urban or rural based on where the household lives in the dataset was used without change.
	Region	Ethiopia's eleven regions are divided into three groups according to their level of development and need for government assistance: the "three metropolises" (Addis Ababa, Harari, and Diredewa); the "large central" (Tigray, Amhara, Oromia, SNNPR); and the "small peripherals" (Afar, Benshangul-Gumuz, Gambelia, and Somali) [24].
	Community level poverty	The percentage of households in the lowest and poorest quintiles found in the wealth index data was used to calculate the community's level of poverty. classified as high if the percentage of households falling into the poor categories exceeded 50% and as low if the percentage was less than 50%.

Data management and analysis

This study utilized data from the EDHS 2019 provided by the DHS program. The results and independent variables were extracted using the household data (HR) set, and STATA version 14 was employed for recording, extracting, and analyzing the data. To ensure the representativeness of the survey and obtain reliable estimates and standard errors, the data were weighted for sampling probabilities using the weighting factor before performing any statistical analysis.

Multilevel analysis was used to account for both individual and community levels due to the hierarchical structure of the EDHS data, where households are nested within enumeration areas (EAs). This approach addresses the violation of the assumption of independence of observations and equal variance across clusters. The Interclass Correlation Coefficient (ICC), Median Odds Ratio (MOR), and Proportional Change in Variance (PCV) were utilized to measure variance through random effects.

The ICC reveals the variation of shared toilet facilities usage between clusters is calculated as; $ICC = \frac{VC}{VC+3.29} * 100\%$, where; VC= cluster level variance

The MOR is defined as the median value of the odds ratio between the area at the lowest risk and the highest risk of shared toilet facilities usage when randomly picking out two clusters.

$\text{MOR} = e^{0.95\sqrt{VC}}$ Where; VC is the cluster-level variance.

The PCV shows the variation in shared toilet facilities usage among households explained by both individual and community level factors. $PCV = \frac{Vnull-VC}{V\ null} * 100\%$ Where; Vnull = variance of the initial model, and VC = cluster level variance of the next model [25-27].

Generally, four models were fitted in multi-level analysis. The first model was the null model, which simply included the outcome variable and was meant to examine the cluster's variability in shared toilet facility utilization. Household and community-level factors are included in the second and third multilevel models, respectively, while shared toilet facility usage was simultaneously

fitted to both household and community-level variables in the fourth model. The deviation test was used to compare the models, and the model that suited the data the best was the one with the lowest deviance[25-27]. In the multivariable analysis, the associations between dependent and independent variables were presented using adjusted odds ratios and 95% confidence intervals with a p-value of <0.05.

Spatial analysis

The Global Moran’s I statistic was used to assess spatial autocorrelation [28]. The Global Moran's I value ranges from −1 to +1, where a value below 0 indicates negative spatial autocorrelation, and values above 0 indicate positive spatial autocorrelation [28, 29]. Based on sampled clusters, we employed a spherical semivariogram ordinary Kriging-type spatial interpolation technique to forecast the extent of shared toilet facilities in Ethiopia for unsampled areas. The input for spatial prediction was the percentage of shared restrooms in each cluster. To determine the locations of shared restroom clusters, Bernoulli-based model spatial scan statistics were applied. [30]. To suit the Bernoulli model, the houses without shared toilets were taken as controls, and the scanning window that moves over the research region with shared toilet facilities was taken as a case.

RESULTS

Sociodemographic characteristics of the study participants

This study included a total of 7,770 households. Among them, males constituted more than three-fourths (6,046 or 77.81%) of the household heads. The majority of participants (72.19%) lived in rural areas, and nearly half (3,785 or 48.71%) of the household heads had no formal education. As the age of household heads increased from 13-30 years to above 57 years, the use of shared toilet facilities decreased from 20.35% to 6.9%, respectively. More than two-thirds (69.09%) of households in metropolitan regions and about one-fourth (28.95%) of household heads in urban residences used shared toilet facilities **[Table 3].**

Table 3: Socio-demographic characteristics and shared toilet usage in Ethiopia, mini 2019 EDHS.

**Variables**	**Categories**	**Shared toilet facilities**		**Total weighted frequency (%)**
		**Yes (%) n = 819 (10.5)**	**No (%) n = 6,951 (89.5)**	
Age of household head (years)	15–24	184 (20.35)	722 (79.65)	906 (11.66)
	25–40	370 (11.40)	2875 (88.60	3245 (41.75)
	41–54	136 (7.74)	1616 (92.26)	1751 (22.53)
	≥55	129 (6.92)	1739 (93.08)	1868 (24.05)
Sex of household head	Male	501 (8.28)	5545 (91.72)	6,046 (77.81)
	Female	318 (18.470	1406 (81.53)	1,724 (22.19)
Educational attainment of household head	No education	228 (6.01)	3,556 (93.99)	3,785 (48.71)
	Primary education	244(8.79)	2,530 (92.21)	2,775(35.71)
	Secondary & above	348 (28.71)	863 (71.29)	1,211 (15.58)
Household family size	1-3	49 8 (17.96)	2141 (82.04)	2,610 (33.59)
	4-6	249 (7.35)	3140 (92.65)	3,889 (43.61)
	7 & above	101 (5.72)	1670 (94.28)	1,771 (22.80)
Wealth index	Poor	60 (2.01)	2,929 (97.99)	2,989 (38.47)
	Middle	36 (2.35)	1,516 (97.65)	1,553 (19.98)
	Rich	723 (22.39)	2,506 (77.61)	3,229 (41.55)
**Community level variables**				
Residence	Urban	625 (28.95)	1,535 (71.05)	2,161 (27.81)
	Rural	193 (3.45)	5,415 (96.55)	5,609 (72.19)
	Metropolis	173 (69.09)	77 (30.91)	250 (3.22)
Region	Large centrals	530 (7.68)	6,373 (92.32)	6,904 (88.85)
	Small periphery	116 (18.82)	500 (81.18)	615 (7.93)

Community poverty	Low	711 (15.43)	3,898 (84.57)	4,610 (59.33)
level	High	108 (3.41)	3,052 (96.59)	3,160 (40.67)

The magnitude of shared sanitation facilities in Ethiopia

In the EDHS 2019, the magnitude of shared sanitation facilities among households in Ethiopia was 10.5% (95% CI: 9.88, 11.24). Of these, more than two-thirds (69.5%) used shared pit latrines with slabs, while only 0.6% used shared sanitation facilities connected to a flush-piped sewer system. The highest prevalence of shared toilet facilities was observed in Addis Ababa (70.2%), whereas the lowest was in the Southern Nations, Nationalities, and Peoples' Region (2.4%) **(Fig. 1)**.

Factors associated with shared or limited access to improved sanitation service in Ethiopia

Since the models were nested, log-likelihood and deviance tests were performed for model comparison. The III-level binary logistic regression model was chosen because it had the largest LR (-1438) and the smallest deviance test result (2,876). The random effects of ICC, PCV, and MOR were evaluated.

The difference at the cluster level was responsible for almost 79% of the variability in shared sanitation facilities among sample families, according to the ICC in the null model. A household from a cluster with a high risk of domestic violence had 28.75 times higher odds of having shared toilet facilities than a household from a cluster with a lower risk of shared toilet facilities, according to the MOR value in the null model (28.75), on the other hand, if you randomly selected two households from two different clusters. This demonstrated the considerable variation in the use of communal restrooms among various populations.

Additionally, the PCV valve in the final model demonstrated that characteristics at both the individual and community levels simultaneously explained nearly 76% of the variation in shared toilet facilities among study families**[Table 4].**

Based on the results from Model III, there was a positive association between improved shared sanitation facilities and several factors such as individuals with higher educational attainment and from greater household wealth, those living in urban areas and metropolitan regions.

As the age of household heads increased to 15–24 years, 25–40 years, and over 55 years, the odds of using shared toilet facilities decreased by 33%, 46%, and 52%, respectively [AOR = 0.66; 95% CI: 0.50, 0.89], [AOR = 0.54; 95% CI: 0.37, 0.79], and [AOR = 0.48; 95% CI: 0.33, 0.71]. The odds of using shared toilet facilities were doubled among household heads with above-primary education [AOR = 2.43; 95% CI: 1.80, 3.28].

Households with a middle or rich wealth index were 2 and 6 times more likely to use shared toilet facilities compared to poor households [AOR = 2.32; 95% CI: 1.35, 3.96] and [AOR = 6.23; 95% CI: 3.84, 10.11], respectively. Individuals living in urban areas were 7.6 times more likely to use shared toilet facilities compared to those in rural areas [AOR = 7.60; 95% CI: 3.47, 16.67]. Residents of metropolitan regions were 26 times more likely, and those in small periphery regions were 5 times more likely to use shared toilet facilities compared to those in large central regions [AOR = 25.83; 95% CI: 10.1, 66.3] and [AOR = 5.01; 95% CI: 2.40, 10.48], respectively[**Table 4**].

Table 4: Multilevel analysis of factors associated with shared toilet facilities usage among households in Ethiopia.

Variables	Categories	Null model	Model I<br>AOR [95% CI]	Model II<br>AOR [95% CI]	Model III<br>AOR [95% CI]
Age of household head (years)	15–24		1.00	-----------	1.00
	25–40		0.67 [0.50, 0.91]*	-----------	**0.66 [0.50, 0.89]***
	41–54		0.56 [0.38, 0.81]**	-----------	**0.54 [0.37, 0.79]***
	≥55		**0.49 [0.34, 0.72]*****	-----------	**0.48 [0.33, 0.71]*****
Sex of household head	Male		1.00	-----------	1.00
	Female		1.17 [0.86, 1.37]	-----------	1.23 [0.92, 1.47]
Educational attainment of household head	No education		1.00	-----------	1.00
	Primary education		1.36 [0.99,1.78]	-----------	1.31 [0.98, 1.73]
	Secondary& above		**2.54 [1.88, 3.44]****	-----------	**2.43 [1.80, 3.28]***
Household family size	1-3		1.00	-----------	1.00
	4-6		0.75 [0.61, 1.03]	-----------	0.76 [0.60, 1.01]
	7 & above		0.88 [0.62, 1.26]	-----------	0.97 [68, 1.38]
Wealth index	Poor		1.00	-----------	1.00
	Middle		2.66 [1.55, 4.56]**	-----------	**2.32 [1.35, 3.96]***
	Rich		**10. 25 [6.39, 16.41]****	-----------	**6.23 [3.84, 10.11**]***
**Community level variables**					
Residence	Urban		-----------	**14.6 [6.61, 32.21] *****	**7.60 [3.47, 16.67]*****
	Rural		-----------	1.00	1.00
Region	Metropolis		-----------	28.97 [11.21, 74.87]***	**25.83 [10.1, 66.3] *****
	Small periphery		-----------	**4.51 [2.14, 9.49]*****	**5.01 [2.40, 10.48] *****
	Large central		-----------	1.00	1.00
Community poverty usage	Low		-----------	1.00	1.00
	High		-----------	0.23 [0.11, 0.49]	0.65 [0.29, 1.39]
**Random effect**					
	Variance	12.5 [8.99, 17.46]	6.75 [4.84, 9.41]	3.27 (2.34, 4.55)	3.05 [2.17, 4.29]
	ICC	0.79 [0.73, 0.84]	0.67 [0.59, 0.74]	0.50 [0.42, 0.58]	0.48 [0.40, 0.57]
	MOR	28.75	11.80	5.57	5.25
	PCV	Reff	0.46	0.74	0.76
**Model comparison**					
	Log-likelihood	-1654	-1515	-1544	-1438
	Deviance	3.308	3,030	3,088	2,876
	Mean VIF	---	1.59	2.18	1.96
** = P-value < 0.05, ** = Pvalue < 0.01, *** = Pvalue < 0.001*					
ICC = Inter cluster correlation coefficient, MOR =Median odds ratio, PCV= proportional change in variance. AOR=adjusted odds ratio; CI= confidence intervale, VIF=Variance Inflation Factors					

Spatial analysis of shared toilet facilities utilization among households in Ethiopia

Spatial autocorrelation analysis of shared toilet facilities

Ethiopia's shared re[illegible]om spatial autocorrelation data revealed a strong positive spatial autocorrelation throughout the nation's regions. It was discovered to be grouped with the value of the Global Moran's Index: 1.78 with (p< 0.001) (**Fig. 2**).

Hotspot Analysis of shared toilet facilities among household in Ethiopia

The Getis-Ord Gi* hotspot analysis showed that shared toilet facilities were more practised in Addis Ababa, and Di[illegible]Dawa, whereas the SNNPR (South Nations Nationalities and People's Region), and Beneshangul Gumuz regions were the cold spot areas (**Fig. 3**).

Significant windows and SaTscan analysis of shared toilet facilities among households in Ethiopia

The SaTScan analysis of shared toilet facilities among households in Ethiopia identified 86 primary clusters and 29 secondary clusters. The primary clusters were centered at coordinates 8.771915 N, 40.335915 E, with a radius of 206.97 km, and were located in Addis Ababa, northern parts of Oromia, southern parts of Amhara and Afar, and Dire Dawa. Households within these primary clusters were 5 times more likely to use shared toilet facilities compared to those outside these clusters (RR = 5.0, P-value < 0.001) (**Table 5, Fig.4).**

Table 5: Significant spatial clusters of shared toilet facilities among households in Ethiopia using 2019 mini-EDHS.

**Clusters**	**Enumeration areas (clusters) detected**	**Coordinate/ radius**	**Population**	**Cases**	**RR**	**LLR**	**P-value**
1[ry] (86)	105, 88, 28, 41, 102, 106, 42, 127, 40, 104, 69, 90, 101, 43, 103, 108, 272, 269, 268, 271, 110, 280, 278, 273, 279, 270, 267, 264, 266, 277, 305, 275, 276, 265, 263, 256, 274, 304, 258, 257, 261, 260, 262, 50, 259, 303, 281, 296,	8.771915 N, 40.335915 E / 206.97 km	2447	851	5.04	181.4 - 489.78	<0.001

	287, 68, 282, 302, 286, 284, 288, 283, 111, 294, 285, 291, 292, 293, 295, 297, 290, 289, 175, 231, 233, 246, 244, 232, 243, 237, 234, 236, 301, 298, 235, 242, 247, 241, 240, 253, 238, 239						
2nd (29)	281, 282, 284, 283, 287, 285, 286, 288, 296, 291, 297, 292, 294, 290 , 289, 295, 302, 264, 273, 267, 263, 270, 276, 265, 275, 266, 258, 271,	10.589922 N, 34.352539 E / 88.60 km	505	245	2.28	7.0-181.3	<0.001

Kiringing interpolation of shared toilet facilities among households in Ethiopia

The Kriging interpolation method for shared toilet facilities among households in Ethiopia indicated that high-risk areas, such as Addis Ababa and Dire Dawa, had predicted usage rates ranging from 50% to 61%. Conversely, the lowest predicted usage rates were observed in the SNNPR, Gambela, Beneshangul Gumuz, and Oromia regions, ranging from 0% to 12% (**Fig. 5)**

DISCUSSION

This study was conducted to assess the magnitude of improved shared toilet facilities and their determinants among households in Ethiopia. Based on this, the prevalence of improved shared toilet facilities in Ethiopia was 10.5% (95% CI: 9.88, 11.24). This is higher than a study by the WHO and UNICEF Joint Monitoring Program in Ethiopia (7%) [7]. It is also higher than in Yemen (4%) and Eritrea (5%), but lower than the global estimate reported in meta-analyses (67%) [4], in Kenya (21%), Ghana (57%), and Uganda (14%) [7]. This variation may be attributed to the different community initiative programs that employ more effective approaches to reducing unimproved sanitation practices and achieving desired sanitation outcomes [18, 31].

In this study, as the age of household heads increases, the usage of shared toilet facilities among households decreases. This is consistent with a study conducted in India, which found that unimproved sanitation practices decrease among older household members [32]. This trend may reflect that as individuals age, they may experience disabilities or incontinence, making it more challenging to use outdoor or shared sanitation facilities [32]. Another factor could be that older individuals, on average, may have reduced mobility and greater difficulty moving freely outside their homes to access sanitation facilities. Additionally, elderly people might be more concerned about their weakened immune systems and th[illegible]creased risk of diseases associated with unimproved sanitation

In this study, as the educational status of household heads increased, the odds of using shared toilet facilities also increased. This is because one-third (32.23%) of households in Ethiopia use open defecation[3] which is more prevalent in less socioeconomic regions. Individuals with at least a formal education are more likely to use shared toilet facilities, which generally have fewer severe consequences for health, safety, and dignity compared to open defecation [1]. Furthermore, educated household heads are more likely to recognize the importance of having sanitation facilities, even if they are limited or shared [33, 34]. According to the World Health Organization

report, education can raise the community's demand for better sanitation facilities [35]. Moreover, a higher level of education enhances awareness and fosters positive attitudes toward choosing relatively more upgraded sanitation facilities [36]. Specifically, educated women are more likely to prefer safe and sanitary facilities that offer privacy and maintain good quality during their menstrual cycle [37]. Moreover, well-educated households with higher incomes had better access to upgraded sanitation facilities [38].

Households that have a rich wealth index are more likely to use the shared toilet as compared to poor households. This is in line with Ethiopia [4], Nigeria [39], and [illegible]anna [40]. This might be because the majority of shared toilet facilities are found in urban areas with a good income-earning population group as compared to open defecation practices that have been taking place in rural areas of low-income countries [41]. In addition, poor households may not have enough place and capacity to construct private and shared toilets and their only option is open defecation. Furthermore, poor households found in developing countries are mainly housed in slums that lack essential infrastructure [37].

The study also found that individuals living in rural households were less likely to use improved shared toilet facilities compared to those in urban areas. This finding is consistent with other research indicating that shared toilet facilities are less common in rural areas, where open defecation remains prevalent, affecting 37% of the rural population in Ethiopia [42]. This may be because many governments do not prioritize rural sanitation in their national agendas, often lacking progressive budgetary support as well as essential legislative and institutional reforms [43]. Besides, the previous study findings suggested that rural households' willingness to pay for 'improved' latrines is minimal [44]. Moreover, rural households are uneducated, and they believe that open defecation is a routine sanitation service [34]. On the other side, sanitation is an investment, and peri-urban inhabitants made the effort to get improved toilet facilities because they had relatively consistent income to support their planning.

The other finding showed that households who live in large central regions were less likely to use shared toilet facilities as compared to metropolitan cities. The spatial analysis result also showed that shared toilet facilities were more commonly practiced in Addis Ababa and Dire Dawa, whereas the SNNPR (South Nations Nationalities and People's Region) and Beneshangul Gumuz regions were the cold spots. This is in line with a study in Mozambique that shows that shared toilet facilities are increasingly common in low-income countries in rapidly growing urban areas[1]. Moreover, in Ethiopia, as a result of internal conflict and drought, informal settlements and refugee camps that use shared toilet facilities are more common in metropolitan cities. The other possible explanation is that those large Centrals contained rural households that practiced open defecation rather than shared and improved sanitation.

The strengths of this study lie in the use of nationally representative, high-quality standardized data, which allows for generalization at the country level. However, a limitation of the study is that being cross-sectional, it cannot establish cause-and-effect relationships.

Conclusion and recommendation

The prevalence of improved shared sanitation services usage among households in Ethiopia is relatively low. Age, educational attainment of the household head, wealth status, residence, and region were found to be significantly associated factors with improved shared sanitation facilities in Ethiopia. Moreover, there was a non-random spatial distribution of improved shared sanitation services in Ethiopia mainly found in Addis Ababa and Dire Dawa. The government of Ethiopia and other stakeholders should provide economic support to low-income households and rural residents to construct improved shared sanitation facilities. Additionally, policy should emphasize user awareness and practices through financial and education literacy to enhance access to and usage of improved shared sanitation facilities, particularly in rural residences and large central regions. Further qualitative studies are needed to explore the behavioral and socio-cultural factors that may prevent individuals from utilizing improved shared sanitation facilities.

Abbreviations

AOR: Adjusted Odds Ratio, CI: Confidence Interval, CSA: Central Statistical Agency, EDHS: Ethiopian Demographic and Health Survey, HR: Household Record, MOH: Ministry Of Health, OD, Open Defecation, SDG: Sustainable Development Goal.

Declarations

Ethical Approval and Consent to Participate

The study doesn't involve the collection of information from subjects, secondary data analysis was done. Ethical approval and consent to participate are not applicable. Since the study is a secondary data analysis based on DHS data.

Consent for publication

Not applicable

Availability of data and materials

Data were accessed after registration, submission of the request, and permission granted via the link *https://dhsprogram.com/data/new-user-registration.cfm*.

The shapefile data was created by GADM, found here: https://gadm.org/index.html, and was created under this license.

Competing Interests

The authors declare that they have no competing interests.

There are no financial, non-financial, or commercial organizations competing for interests

Funding

No funding was obtained for this study.

Authors' contribution

The conception of the work, design of the work, acquisition of data, analysis, and interpretation of data were done by DGB, and BTA. Data curation, drafting the article, revising it critically for intellectual

content, validation, and final approval of the version to be published was done by BTA and DGB. Both authors read and approved the final manuscript.

Acknowledgments

I would like to thank the “measure DHS program” for providing the data set.

Figure legends

Fig. 1: Magnitude of shared toilet facilities among households in Ethiopia, using the 2019 EDHS.

Fig.2: Spatial autocorrelation analysis of shared toilet facilities among households in Ethiopia using 2019 mini-EDHS. The base map for the shapefile was sourced from: https://gadm.org/

Fig.3: Hotspot analysis of shared toilet facilities among households in Ethiopia using 2019 mini-EDHS. The base map for the shapefile was sourced from: https://gadm.org/ .

Fig. 4: SaTscan analysis of shared toilet facilities among households in Ethiopia using 2019 mini-EDHS.: The base map for the shapefile was sourced from: https://gadm.org/

Fig. 5: Kiringing interpolation of shared toilet facilities among households in Ethiopia using 2019 mini-EDHS.:The base map for the shapefile was sourced from: https://gadm.org/.

References

1. Shiras, T., et al., *Shared Sanitation Management and the Role of Social Capital: Findings from an Urban Sanitation Intervention in Maputo, Mozambique.* Int J Environ Res Public Health, 2018. **15**(10).
2. World Health Organization and UNICEF, *Progress on Drinking Water, Sanitation and Hygiene Update and SDG Baselines 2017*. 2017, WHO, Geneva.
3. Central Statistical Agency (CSA) [Ethiopia] and ICF. 2016, *Ethiopia Demographic and Health Survey 2016*. Addis Ababa, Ethiopia, and Rockville, Maryland, USA: CSA and ICF.
4. Sprouse, L., et al., *Shared sanitation in informal settlements: A systematic review and meta-analysis of prevalence, preferences, and quality.* International Journal of Hygiene and Environmental Health, 2024. **260**: p. 114392.
5. Simiyu, S., *Why shared toilets in informal settlements may pose a serious health risk, available on https://theconversation.com/why-shared-toilets-in-informal-settlements-may-pose-a-serious-health-risk-94339.* 2018.
6. Abebe, T.A. and G.T. Tucho, *Open defecation-free slippage and its associated factors in Ethiopia: a systematic review.* Syst Rev, 2020. **9**(1): p. 252.
7. World Health Organization, *Progress on drinking water, sanitation and hygiene: 2017 update and SDG baselines.* 2017.
8. Evans, B., et al., *Limited services? The role of shared sanitation in the 2030 agenda for sustainable development*. 2017, IWA Publishing.
9. Spears, D., A. Ghosh, and O. Cumming, *Open defecation and childhood stunting in India: an ecological analysis of new data from 112 districts.* PloS One, 2013. **8**(9): p. e73784.

10. John, J.R., et al., *Determinants of early initiation of breastfeeding in Ethiopia: a population-based study using the 2016 demographic and health survey data.* BMC Pregnancy Childbirth, 2019. **19**(1): p. 69.

11. Debela, T.H., et al., *Fecal contamination of soil and water in sub-Saharan Africa cities: The case of Addis Ababa, Ethiopia.* Ecohydrology & Hydrobiology, 2018. **18**(2): p. 225-230.

12. Gebru, T., M. Taha, and W. Kassahun, *Risk factors of diarrhoeal disease in under-five children among health extension model and non-model families in Sheko district rural community, Southwest Ethiopia: a comparative cross-sectional study.* BMC Public Health, 2014. **14**(1): p. 1-6.

13. Liu, L., et al., *Global, regional, and national causes of child mortality: an updated systematic analysis for 2010 with time trends since 2000.* The Lancet, 2012. **379**(9832): p. 2151-2161.

14. UNICEF, *Sanitation issue.* Addis Ababa: Ethiopian Newsletter, 2004.

15. United Nations, *United Nations Department of Economic and Social Affairs, Transformational benefits' of ending outdoor defecation: Why toilets matter available on,https://www.un.org/development/desa/en/news/sustainable/world-toilet-day2019.html.* 2018.

16. World Health Organization, *Progress on drinking water, sanitation, and hygiene: 2017 update and SDG baselines. Available at: https://iris.who.int/bitstream/handle/10665/258617/9789241512893eng.pdf?sequence=1.* 2017.

17. Research, B., *Outcome evaluation of Community-Led Total Sanitation and Hygiene (CLTSH) Program in Ethiopia from 2012-2015*. 2016, UNICEF Addis Ababa.

18. Kar, K. and R. Chambers, *Handbook on community-led total sanitation.* 2008.

19. Leshargie, C.T., et al., *Household latrine utilization and its association with educational status of household heads in Ethiopia: a systematic review and meta-analysis.* BMC Public Health, 2018. **18**(1): p. 1-12.

20. Corsi, D.J., et al., *Demographic and health surveys: a profile.* International journal of epidemiology, 2012. **41**(6): p. 1602-1613.

21. Worldometer, *African Countries by Population (2024) - Worldometer https://www.worldometers.info/population/countries-in-africa-by-population/*. 2024.

22. Ethiopian Public Health Institute, a.I., *Ethiopia mini demographic and health survey 2019: key indicators. J Chem Information Model. 53:1689–1699.* 2019.

23. Croft, T., M. Aileen, and C. Marshall, *Allen,(2018).* Guide to DHS statistics.

24. Teshale, A.B. and G.A. Tesema, *Magnitude and associated factors of unintended pregnancy in Ethiopia: a multilevel analysis using 2016 EDHS data.* BMC pregnancy and childbirth, 2020. **20**(1): p. 329-329.

25. Liyew, A.M. and A.B. Teshale, *Individual and community level factors associated with anemia among lactating mothers in Ethiopia using data from Ethiopian demographic and health survey, 2016; a multilevel analysis.* BMC Public Health, 2020. **20**: p. 1-11.

26. Merlo, J., et al., *A brief conceptual tutorial of multilevel analysis in social epidemiology: linking the statistical concept of clustering to the idea of contextual phenomenon.* Journal of Epidemiology & Community Health, 2005. **59**(6): p. 443-449.

27. Merlo, J., et al., *A brief conceptual tutorial on multilevel analysis in social epidemiology: interpreting neighborhood differences and the effect of neighborhood characteristics on individual health.* Journal of Epidemiology & Community Health, 2005. **59**(12): p. 1022-1029.

28. McMillen, D.P., *Geographically weighted regression: the analysis of spatially varying relationships*. 2004, Oxford University Press.

29. exchange, S., *Choosing the value of Moran's I to say the existence of spatial correlation; access date: 22 March 2023. available at: https://gis.stackexchange.com/questions/269013/choosing-value-of-morans-i-to-say existence-of-spatial-correlation.* 2018.
30. Kulldorff, M., *A spatial scan statistic.* Communications in Statistics-Theory and methods, 1997. **26**(6): p. 1481-1496.
31. Abebe, T.A. and G.T. Tucho, *Open defecation-free slippage and its associated factors in Ethiopia: a systematic review.* Systematic reviews, 2020. **9**(1): p. 1-15.
32. Coffey, D., et al., *Revealed preference for open defecation.* Economic & Political Weekly, 2014. **49**(38): p. 43.
33. Paavola, J., *Livelihoods, vulnerability and adaptation to climate change in Morogoro, Tanzania.* Environmental Science & Policy, 2008. **11**(7): p. 642-654.
34. Tumwine, J., et al., *Sanitation and hygiene in urban and rural households in East Africa.* International journal of environmental health research, 2003. **13**(2): p. 107-115.
35. Project, T.B., *EDUCATION AND IMPROVING SANITATION IN DEVELOPING COUNTRIES.* https://borgenproject.org › SEPTEMBER 26, 2016.
36. Wei, F. and V.K. Pillai, *Sanitation in India: Role of women's education.* 2014.
37. Donacho, D.O., G.T. Tucho, and A.B. Hailu, *Households' access to safely managed sanitation facility and its determinant factors in Jimma town, Ethiopia.* Journal of Water, Sanitation and Hygiene for Development, 2022. **12**(2): p. 217-226.
38. Nyambe, S., L. Agestika, and T. Yamauchi, *The improved and the unimproved: factors influencing sanitation and diarrhea in a peri-urban settlement of Lusaka, Zambia.* PloS one, 2020. **15**(5): p. e0232763.

39. Abubakar, I.R., *Exploring the determinants of open defecation in Nigeria using demographic and health survey data.* Science of the total environment, 2018. **637**: p. 1455-1465.

40. Osumanu, I.K., E.A. Kosoe, and F. Ategeeng, *Determinants of open defecation in the Wa municipality of Ghana: empirical findings highlighting sociocultural and economic dynamics among households.* Journal of environmental and public health, 2019. **2019**.

41. Galan, D.I., S.-S. Kim, and J.P. Graham, *Exploring changes in open defecation prevalence in sub-Saharan Africa based on national level indices.* BMC Public Health, 2013. **13**(1): p. 1-12.

42. Beyene, A., et al., *Current state and trends of access to sanitation in Ethiopia and the need to revise indicators to monitor progress in the Post-2015 era.* BMC Public Health, 2015. **15**(1): p. 1-8.

43. World Bank, *Why rural sanitation matters.* https://blogs.worldbank.org, NOVEMBER 14, 2019.

44. Affairs, M.o.F., *Rural Water and Sanitation.* https://www.oecd.org Number 6 · July 2012.

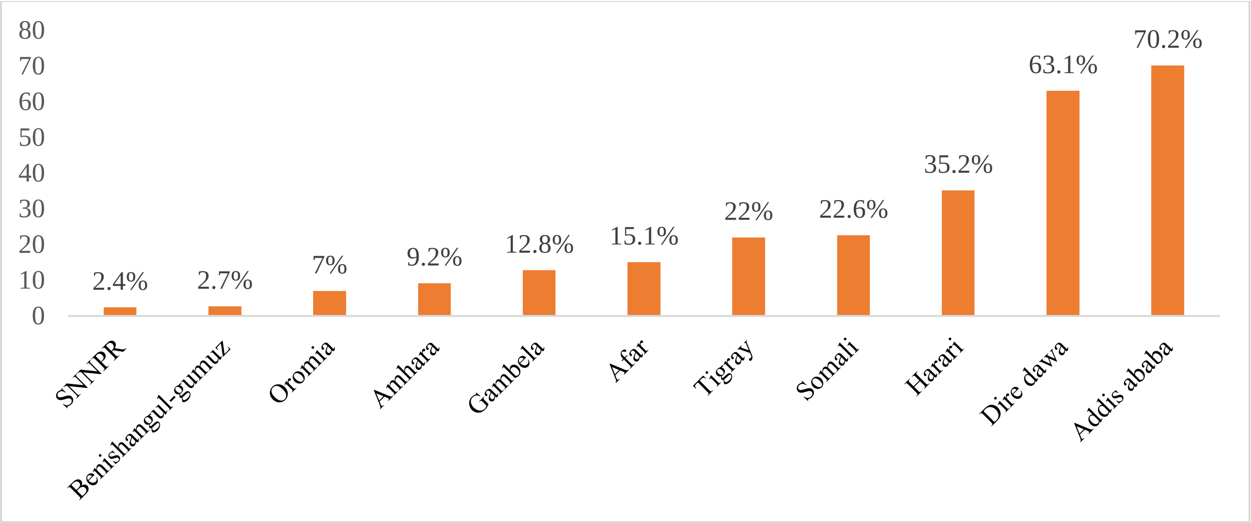

Figure 1: Magnitude of shared toilet facilities among households in Ethiopia, using the 2019 EDHS

Click here to access/download;Figure;Figure 2 Revised.docx

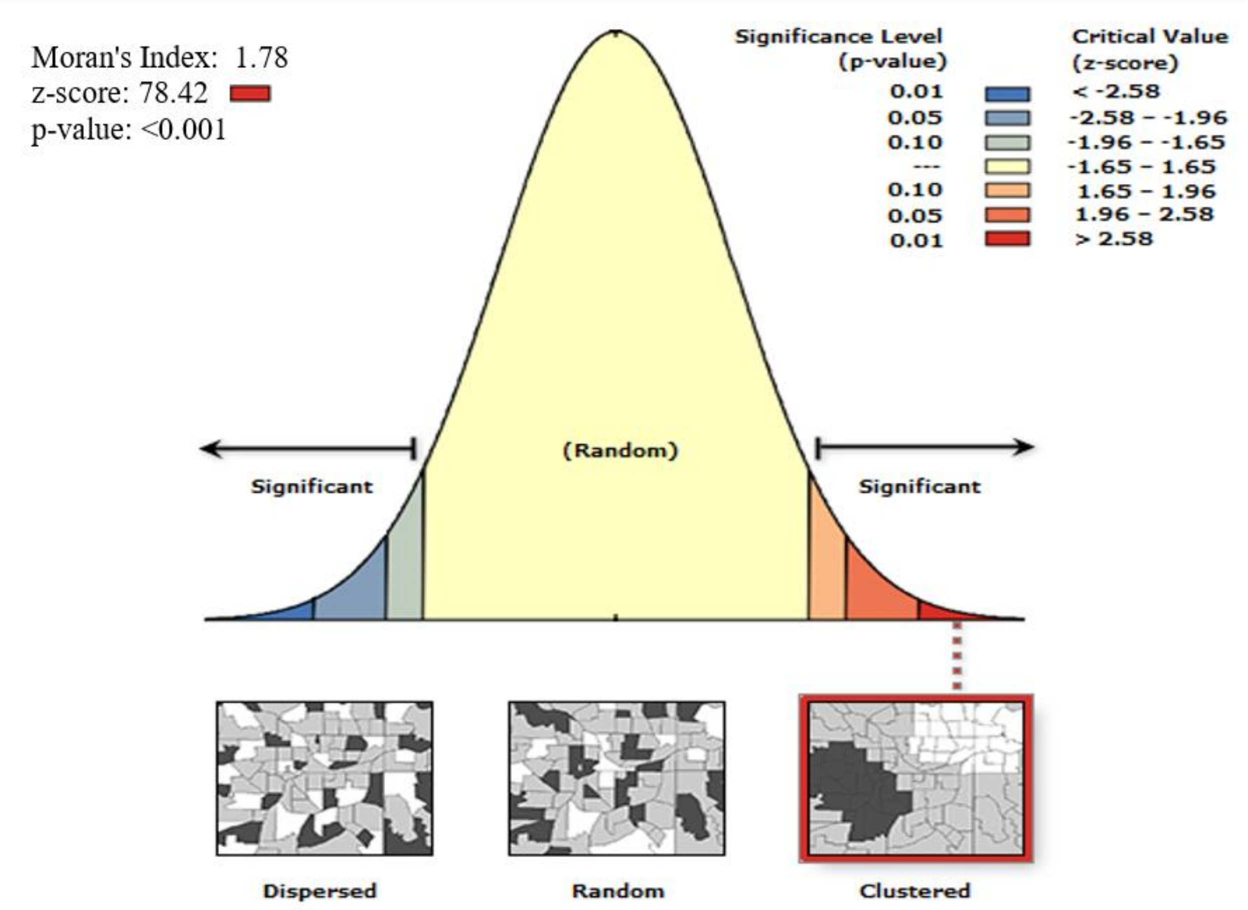

Figure 2: Spatial autocorrelation analysis of shared toilet facilities in Ethiopia

Click here to access/download;Figure;Figure 3 Revised.docx

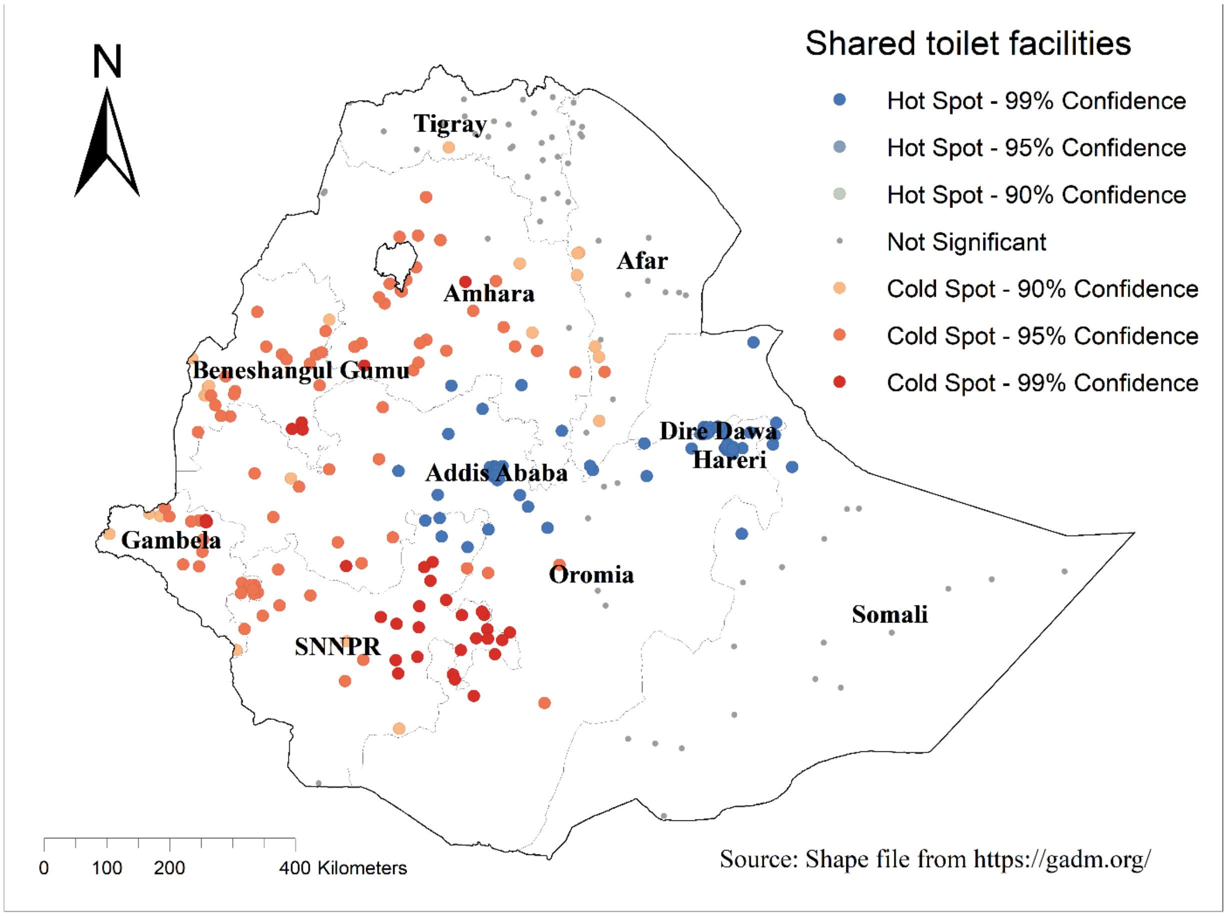

Figure 3: Hotspot analysis of shared toilet facilities among household in Ethiopia

Figure Click here to access/download;Figure;Figure 4 Revised.docx

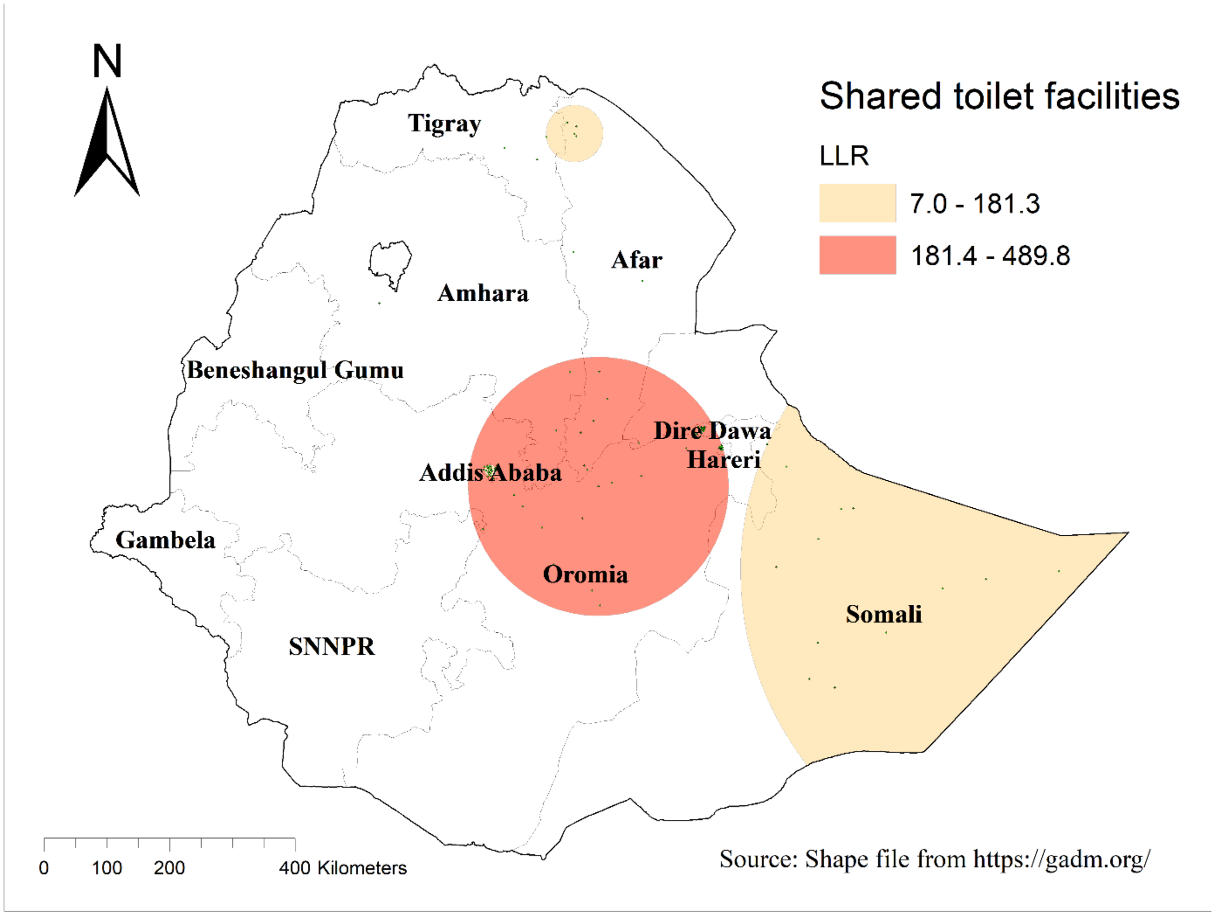

Figure 4: SaTscan analysis of shared toilet facilities among households in Ethiopia using 2019 mini-EDHS.: The base map for the shapefile was sourced from: https://gadm.org/ .

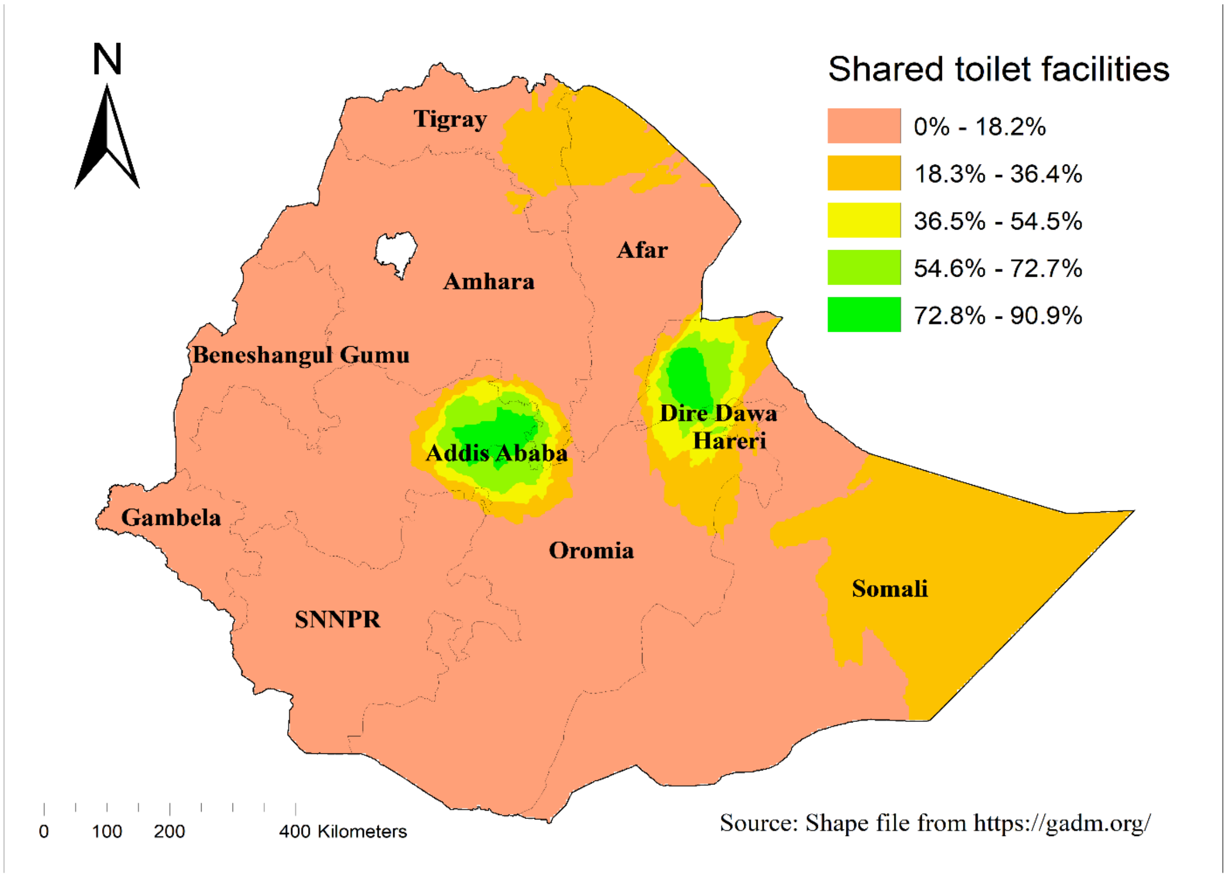

Figure 5: Kiringing interpolation of shared toilet facilities among households in Ethiopia using 2019 mini-EDHS. The base map for the shapefile was sourced from: https://gadm.org/

Spatial distribution and determinants of improved shared ~~toilet~~sanitation facilities among households in Ethiopia: Using 2019 mini-Ethiopian Demographic and Health Survey~~: Multi-level analysis~~

Baye Tsegaye Amelak[1], Daniel Gashaneh Belay [2, 3],

[1]Department of Nursing, College of Medicine and Health Sciences, Debre Markos University, Debre Markos, Ethiopia, Email: bayetsegaye07@gmail.com

[2]Department of Human Anatomy, School of Medicine, College of Medicine and Health Sciences, University of Gondar, Gondar, Ethiopia. Email, danielgashaneh28@gmail.com

[3]Department of Epidemiology and Biostatistics, Institute of Public Health, College of Medicine and Health Sciences, University of Gondar, Gondar, Ethiopia

[*]**Corresponding author**

Baye Tsegaye Amlak (Department of Nursing, College of Health Sciences, Debre Markos University, Debre Markos, Ethiopia. Email: bayetsegaye07@gmail.com

Formatted: Font color: Auto

Formatted: Font color: Auto

Formatted: Font color: Auto

Formatted: Font color: Auto

Formatted: Font color: Auto

Formatted: Font color: Auto

ABSTRACT

Introduction: Limited or shared sanitation services ~~or shared toilet facilities~~ are considered improved sanitation facilities~~-~~, but they are shared between two or more households. Globally, ~~2.3 billion people lack access to improved sanitation facilities, and from these~~ 600 million people use shared toilet facilities~~. In Ethiopia,~~. Although shared ~~toilet~~ facilities ~~usage becomes increasing. Even if shared facilities~~ are not ~~recognized~~classified as improved sanitation due to ~~the possibilities for the spread of infections due to poor hygiene, inaccessible and unsafe,~~potential infection risks, inaccessibility, and safety concerns, this is a significant issue in developing countries like Ethiopia. Evidence on the distribution of shared sanitation services and their determinants in Ethiopia ~~where conflict and internal displacement, sharing toilets allows residents, refugee camps, and informal settlements to access and use toilets.~~is limited. Therefore, this study aimed to assess the ~~magnitude~~extent of shared toilet facilities and their determinants among households in Ethiopia.

Methods: The 2019 Ethiopian Demographic and Health Survey (EDHS) served as the basis for the cross-sectional secondary data analysis~~ that was done. 8,663~~. The analysis included a total of 7,770 households ~~in total,~~from the weighted sample~~, were included.~~. STATA 14 software was used to clean, weigh, and analyze the data. ~~In order to~~ To explore the distribution and determine the factors ~~influencing~~associated with shared ~~toile~~toilet facilities in Ethiopia, ~~a~~both spatial and mixed-effect ~~analysis was~~ analyses were utilized. A p-value of less than 0.05 was used to display the relationships between the dependent and independent ~~and dependent~~ variables~~ using,~~ employing adjusted odds ratios and 95% confidence intervals.

Results: The magnitude of improved shared toilet facility usage sanitation facilities among households in Ethiopia, according to the EDHS 2019, was 9.4610.5% (95% CI: 8.86, 10.09), with the 9.88, 11.24). The prevalence was highest (44.6%) in Dire DawaAddis Ababa at 70.2% and the lowest (2.2%) in the Southern NationNations, Nationalities, and People's. The individual Peoples' Region at 2.4%. Individual-level variables that were statistically significantsignificantly associated with the use of improved shared toilet facility usage werefacilities included being a household head age >57aged 55 years or older [AOR=0.5448; 95% CI;: 0.4233, 0.6971], having secondary and above educational statuseducation or higher [AOR=1.312.43; 95%CI; 1.04, 1.64], having seven and above family members [AOR= 0.55; 95%CI; 0.42, 0.73], and CI: 1.80, 3.28], and belonging to middle or rich wealth status [middle: AOR=2.32; 95% CI: 1.35, 3.96; rich: AOR= 4.886.23; 95% CI; 3.38, 7.06]. On the other hand, community: 3.84, 10.11]. Community-level characteristics that were statisticallysuch as residing in urban areas [AOR=7.60; 95% CI: 3.47, 16.67], the metropolitan region [AOR=25.83; 95% CI: 10.1, 66.3], and periphery regions [AOR=5.01; 95% CI: 2.40, 10.48] were also associated with the use of shared toilet facilities included living in rural households [AOR=0.08; 95%CI; 0.05, 0.14] and large central regions [AOR=0.17; 95%CI; 0.12, 0.26]..

Conclusion: The proportionusage of improved shared toilet facility usage facilities among households in Ethiopia is relatively low. Age, educational attainmentSignificant factors related to the use of household head, household family size, and shared toilet facilities were being 55 years of age or older, possessing secondary or higher education, having a middle or rich wealth status, living in urban areas, and residing in metropolitan or peripheral regions. To improve access to and community-level variables such as residenceutilization of shared sanitation facilities, Ethiopian policy should emphasize user education and region, were found to be significantly associated with itawareness.

Formatted: Pattern: Clear
Formatted: Pattern: Clear
Formatted: Pattern: Clear
Formatted: Pattern: Clear
Formatted: Pattern: Clear
Formatted: Pattern: Clear
Formatted: Pattern: Clear
Formatted: Pattern: Clear
Formatted: Pattern: Clear
Formatted: Pattern: Clear
Formatted: Pattern: Clear
Formatted: Pattern: Clear
Formatted: Pattern: Clear
Formatted: Pattern: Clear
Formatted: Pattern: Clear
Formatted: Pattern: Clear
Formatted: Pattern: Clear
Formatted: Pattern: Clear
Formatted: Pattern: Clear
Formatted: Pattern: Clear
Formatted: Pattern: Clear
Formatted: Pattern: Clear
Formatted: Pattern: Clear
Formatted: Pattern: Clear
Formatted: Pattern: Clear
Formatted: Pattern: Clear
Formatted: Pattern: Clear
Formatted: Pattern: Clear
Formatted: Pattern: Clear
Formatted: Pattern: Clear
Formatted: Pattern: Clear
Formatted: Pattern: Clear

Keywords: Shared toilet, limited sanitationEDHS 2019, determinant, spatial, households, Ethiopia

Introduction

Globally, 2.3 billion people lack access to improved sanitation facilities [1]. From these, 600 million people use limited sanitation services (shared toilet facilities) [1]. World Health Organization (WHO) and United Nations Joint Monitoring Programme (JMP) defined limited sanitation services as improved sanitation facilities shared between two or more households [1, 2].

The number of individuals who practice shared toilet facilities increased from 3 million to 6 million between 2000 and 2015 globally [3]. Whereas in Sub-Saharan African countries people who used shared toilet facilities climbed from 0.64 million to 0.96 million which was 18% of all households [1, 4]. Ethiopia is one of the sub-Saharan African countries that showed increasing shared toilet facilities usage in the same way as other sub-Saharan African countries from 4% to 7% between the years 2000 and 2015 [3].

Shared facilities are not recognized as improved sanitation due to challenges of maintenance, and as they are easy, they can be avenues for the spread of infections due to poor hygienic, inaccessible, and unsafe conditions [5]. These are frequently not well kept, creating unclean conditions that discourage frequent use [2]. Global research has demonstrated the connection between using shared toilet usage and unfavorable health outcomes like helminth infections, diarrhea, enteric fevers, and fecal-oral illnesses [6]. It aids the transmission of microorganisms that cause diarrheal diseases [7], with children being the most vulnerable [8]. Diarrheal disease is the second leading cause of death for children under five worldwide, accounting for 760 000 deaths and 1.7 million morbidities annually [9]. In Africa, diarrhea is one of the main causes of death in under-five children [10], it kills half a million under-five children annually only in Ethiopia [11]. In addition, using a shared toilet puts hundreds of millions of women and children at risk for increased sexual exploitation and a lack of privacy during their menstrual cycle [12] [13]. Poor sanitation is also linked to infections and eye diseases such as trachoma [13].

However, informal settlements are widespread and typically characterized by substandard housing, poverty, and a lack of basic sanitation facilities in developing nations like Ethiopia, where conflict and internal displacement are prominent [13]. Therefore, sharing toilets allows residents who do not have toilets in their homes to access toilets [13]. The Sustainable Development Goal (SDG) target 6.2 states that by 2030, all children shall have access to universal sanitation facilities and

that no child should suffer a disease or pass away from drinking tainted water or coming into contact with the excreta of others [5]. Therefore, providing shared sanitation services could be a crucial first step in achieving the universal sanitation coverage aim set forth in the Sustainable Development Goals [5]. Shared sanitation is the sole option available to millions of people living in densely packed metropolitan areas, particularly in informal settlements, as opposed to open defecation, which has far greater detrimental effects on one's health, safety, and dignity [2]. Furthermore, although the default design of shared toilets is an enhanced sort of sanitation facility, they are regarded as unimproved due to our sharing of them [14]. As a result, they are superior to all other forms of open defecation and other outdated sanitation infrastructure. Ethiopia has prioritized the sanitation program since 1995, when the government wrote public health into the country's National Constitution. Subsequently, in 2005 and 2006, respectively, the Ministry of Health created the National Hygiene and Sanitation Strategy and the National Hygiene and On-Site Sanitation Protocol[15, 16]. However, the magnitude and contributing factors to using shared toilet facilities in Ethiopia are little known [17] and, children's death due to diarrheal disease is common [11]. Therefore, this study aims to answer the following research questions: What was the magnitude of shared toilet facilities in Ethiopia? What are other contributing factors significantly associated with shared toilet facilities? So answering these questions will be valuable for policymakers and program planners as preliminary evidence to plan and decide accordingly.

The World Health Organization (WHO) and the United Nations Joint Monitoring Programme (JMP) define limited sanitation services as improved sanitation facilities that are shared between two or more households [1, 2]. Globally, 2.3 billion people lack access to improved sanitation facilities [2]. From these, 600 million people use shared toilet facilities [2].

Shared sanitation is the only option available to millions of people living in densely packed urban areas, especially in informal settlements. This is preferable to open defecation, which has far more

severe negative impacts on health, safety, and dignity [1]. Furthermore, while shared toilets are designed to be an improved type of sanitation facility, they are considered unimproved because they are shared among multiple users [3].

A meta-analysis of global studies on shared sanitation in informal settlements estimated that the overall prevalence of shared sanitation was 67%. Users' preferences for using shared facilities were influenced by factors such as cleanliness, affordability, safety, privacy, structural quality, and accessibility[4]. However, informal settlements are widespread and often characterized by substandard housing, poverty, and a lack of basic sanitation facilities. This is especially true in developing nations like Ethiopia, where conflict and internal displacement are prevalent [5]. Therefore, sharing toilets provides residents who lack private toilets in their homes with access to sanitation facilities [5].

In Sub-Saharan African countries, the number of households using shared toilet facilities increased from 0.64 million to 0.96 million, representing 0.08% of all households [2, 6]. Similarly, in Ethiopia, the use of shared toilet facilities rose from 4% to 7% between 2000 and 2015 [7]. Shared facilities are not regarded as an improvement in sanitation because they frequently encounter maintenance issues. Furthermore, their accessibility can lead to the spread of infections due to poor hygiene, limited accessibility, and unsafe conditions [8]. These are frequently not well kept, creating unclean conditions that discourage frequent use [1]. Global research has shown a connection between the use of shared toilets and adverse health outcomes such as helminth infections, diarrhea, enteric fevers, and fecal-oral diseases [9]. It aids the transmission of microorganisms that cause diarrheal diseases [10], with children being the most vulnerable [11]. Diarrheal disease is the second leading cause of death for children under five worldwide, accounting for 760,000 deaths and 1.7 million morbidities annually [12]. In Africa, diarrhea is one of the main causes of death in under-five children [13], It causes the deaths of half a million children under the age of five annually in Ethiopia alone [14]. Moreover, using shared toilets puts

hundreds of millions of women and children at greater risk of sexual exploitation and a lack of privacy during their menstrual cycles [15] [5]. Poor sanitation is also associated with infections and eye diseases, such as trachoma[5].

Sustainable Development Goal (SDG) target 6.2 aims to ensure that by 2030, all children will have access to universal sanitation facilities [8] and that no child should suffer from disease or die due to contaminated water or contact with human waste [16]. Therefore, providing shared sanitation services could be a crucial initial step toward achieving the universal sanitation coverage goal set out in the Sustainable Development Goals [8]. Since 1995, Ethiopia has prioritized its sanitation program, following the inclusion of public health in the country's National Constitution. Subsequently, in 2005 and 2006, the Ministry of Health developed the National Hygiene and Sanitation Strategy and the National Hygiene and On-Site Sanitation Protocol, respectively[17, 18]. However, the magnitude and contributing factors to using shared toilet facilities in Ethiopia are little known [19]. Therefore, this study aims to address the following research questions: What is the magnitude and spatial distribution of improved shared sanitation facilities in Ethiopia? What factors are associated with improved shared sanitation facilities? Answering these questions will provide valuable insights for policymakers and program planners, helping them allocate resources more effectively, design targeted interventions, and develop relevant policies.

Methodology

Study design, setting, and data source

This study ~~used~~ utilized data from the recent Ethiopian Demographic and Health Survey ~~data~~ (EDHS 2019~~)~~), which ~~were~~was collected using a community-based cross-sectional study design. ~~The~~ Since 1984, the DHS ~~collects~~has gathered a ~~wide~~broad range of objective and self-reported data ~~in~~across more than 99 countries~~ worldwide, since 1984.~~. Key advantages of the DHS include

high response rates, national coverage, high qualityrigorous interviewer training, standardized data collection procedures across countries, and consistent content over time [18][20].

With 1.1 million square kilometers under its belt and an expected 114,963,588132,059,767 inhabitants in 20212024, Ethiopia is the second most populated country in Africa [19].next to Nigeria [21]. Ethiopia has two city administrations (Addis Ababa and Dire Dawa) and nine regions (Tigray, Afar, Amhara, Oromia, Benishangul Gumuz, Somalia, South Nation Nationalities and Peoples of Ethiopia (SNNP), Gambelia, and Harari) with a federal decentralized administrative structure. In EDHS, stratified, two stage cluster sampling methods were applied. By dividingTo ensure survey precision was comparable across regions, the sample allocation was carried out through equal distribution, with 35 enumeration areas (EAs) selected from each region into itsof the three larger regions: Amhara, Oromia, and the Southern Nations, Nationalities, and Peoples' Region (SNNPR). Additionally, 25 EAs were selected from each of eight other regions. In the first stage, a total of 305 EAs (93 in urban areas and 212 in rural components, it was stratified. Following a areas) were chosen with probability selection process commensurate with theproportional to EA size of each Enumeration region (EA), 645 EAs (202 in the urban region) were selected in the first stage. The. In the second stage was the careful selection of,, an average of 25–30 households were carefully selected from each EA based on average, 25–30 homes. The specifics of the study's design and environment can be found elsewhere [14]the 2019 Ethiopian Population and Housing Census (EPHC) frame[22].

Study population

The study population consisted of all houses that had theirunimproved sanitation facilitiesservices evaluated during the 2019 mini EDHS survey. Ultimately, a weighted sample of (7,561). This includes, shared improved sanitation services (1,276), unimproved sanitation facilities (3,442), and open defecation (OD) (2,843).

Out of the 8,663 households included in the 2019 mini EDHS survey, 1,102 households had improved but not shared sanitation facilities and were excluded from the analysis. Ultimately, a sample of 7,561 households (weighted to 7,770) was included in the analysis.

Study variables

The outcome variable of the study was shared/ limited sanitation service which means households with either one type of improved sanitation facility but shared with other households ~~[20]~~[23] **(Table 1).**

Table 1: Questions to measure improved ~~and~~but shared/ limited sanitation service.

~~Improved~~**Outcome variable (Shared/ limited sanitation ~~facility (Having yes for either of the following~~service)**	~~Shared/ limited sanitation service~~ **Control group/comparators**
Did the toilet have flush/pour flush to the piped sewer system?	~~***Does either of this improved toilet share with other households?***~~<br>✓ ~~***If the answer is yes for the above question, it is shared/limited***~~Unimproved sanitation ~~service.~~services such as<br>o Pit latrine without slab/open pit<br>o Bucket toilet<br>o Hanging toilet/latrine<br>o Other<br>✓ Open defecation: no facility/bush/field
Did the toilet have flush/pour flush to the septic tank?	
Did the toilet have a flush to somewhere else?	
Did the toilet have flush/pour flush to the pit latrine?	
Did the toilet have a flush, *don't know where.*	
Was the toilet Ventilated Improved Pit (VIP) latrine?	
Was the toilet pit latrine with a slab?	
Was the toilet composting toilet?	
If a household uses any of the listed above-improved sanitation facilities but shares them with other households.	

The study's predictor variables were ~~classified as~~categorized into individual-level variables, ~~including~~which included the age, sex, and educational attainment of household heads, as well as factors ~~like~~such as the household wealth index, ~~household~~ family size, and household size. ~~This study examined~~Additionally, community-level characteristics such as residence location ~~of~~

Formatted Table
Formatted: Font: Bold
Formatted: Font: Bold
Formatted: Font: Bold
Formatted: Font: Not Bold, Not Italic
Formatted Table
Formatted Table
Formatted: List Paragraph, Bulleted + Level: 1 + Aligned at: 0" + Indent at: 0.25"
Formatted: Font: Not Bold, Not Italic

~~residence~~, region, and community poverty~~.~~ were examined. After ~~evaluating~~ assessing the normal distribution of the aggregated community ~~components' normal distribution~~ components using a histogram and the Shapiro-~~Wilks~~Wilk test, the data were ~~recoded~~recorded using the appropriate measures of central tendency ~~measure~~ **(Table 2).**

Table 2: ~~***a***~~***A list of the study's variables along with an explanation of each measurement***

Level	Variables	Measurements
Individual level variables	Age	The age of the participants was categorized as ~~13-30, 31~~15-24, 25-40, 41-~~56, >57~~54, >55
	Sex	Sex of the household head as male and female
	Education level	Educational attainment is categorized as uneducated, primary, secondary, and above
	Family size	Categorized as 1-3, 4-6, and 7 and above.
	Wealth index	A wealth index categorized as poorest, ~~poorer,~~ intermediate, ~~richer~~richest, and wealthiest in the DHS data collection was developed using principal components analysis and included in the datasets. We classified it into three categories for this study: middle class, rich (including wealthier ~~and richest~~), and poor (including poorer and poorest).
Community level variables	Residency	Urban or rural based on where the household lives in the dataset was used without change.
	Region	Ethiopia's eleven regions are divided into three groups according to their level of development and need for government assistance: the "three metropolises" (Addis Ababa, Harari, and Diredewa); the "large central" (Tigray, Amhara, Oromia, SNNPR); and the "small peripherals" (Afar, Benshangul-Gumuz, Gambelia, and Somali) ~~[21].~~ [24].

Formatted: Font: Bold

Formatted: Font: Bold

Formatted Table

	Community level poverty	The percentage of households in the lowest and poorest quintiles found in the wealth index data was used to calculate the community's level of poverty. classified as high if the percentage of households falling into the poor categories exceeded 50% and as low if the percentage was less than 50%.

Data management and analysis

Formatted: Space Before: 0 pt

~~Based on EDHS 2019~~ This study utilized data from the EDHS 2019 provided by the DHS ~~measure~~ program~~, this study was carried out.~~. The ~~result~~results and ~~the~~ independent variables were extracted using the household data (HR) set~~.~~, and STATA version 14 was ~~used~~employed for recording, extracting, and analyzing the data. ~~In order to recover~~To ensure the ~~survey's~~ representativeness of the survey and obtain ~~a trustworthy estimate~~reliable estimates and standard ~~error~~errors, the data were weighted for ~~the~~ sampling probabilities using the weighting factor ~~prior to~~ before performing any statistical analysis.

Multilevel analysis~~, which takes into~~ was used to account for both ~~the~~ individual and community levels~~, was utilized~~ due to ~~evaluate~~ the ~~clustering effect because the EDHS data has a~~ hierarchical structure ~~in which~~of the EDHS data, where households are nested within ~~an EA, thus defying~~enumeration areas (EAs). This approach addresses the violation of the assumption of independence of observations and equal variance across clusters. The Interclass Correlation Coefficient (ICC), Median Odds Ratio (MOR), and Proportional Change in Variance (PCV) were utilized to ~~construct a~~ measure ~~of~~ variance ~~utilizing~~through random effects.

Formatted: Justified

The ICC reveals the variation of shared toilet facilities usage between clusters is calculated as; $ICC = \frac{VC}{VC+3.29} * 100\%$, where; VC= cluster level variance

The MOR is defined as the median value of the odds ratio between the area at the lowest risk and the highest risk of shared toilet facilities -usage when randomly picking out two clusters.

$\mathrm{MOR} = e^{0.95\sqrt{VC}}$ Where; VC is the cluster-level variance.

The PCV shows the variation in shared toilet facilities -usage among households explained by both individual and community level factors. $PCV = \frac{Vnull - VC}{V\ null} * 100\%$ Where; Vnull = variance of the initial model, and VC = cluster level variance of the next model ~~[22-24]~~[25-27].

Generally, four models were fitted in multi-level analysis. The first model was the null model, which simply included the outcome variable and was meant to examine the cluster's variability in shared toilet facility~~–~~ utilization. Household and community~~-~~-level factors are included in the second and third multilevel models, respectively, while shared toilet facility usage was simultaneously fitted to both household and community~~-~~-level variables in the fourth model. The deviation test was used to compare the models, and the model that suited the data the best was the one with the lowest deviance~~[22-24]~~[25-27]. In the multivariable analysis, the associations between dependent and independent variables were presented using adjusted odds ratios and 95% confidence intervals with a p-value of <0.05.

Spatial analysis

The Global Moran's I statistic was used to assess spatial autocorrelation ~~[25].~~[28]. The Global Moran's I value ranges from −1 to +1, where a value below 0 indicates negative spatial autocorrelation, and values above 0 indicate positive spatial autocorrelation ~~[25, 26]. On the basis of sampled clusters, we employed a spherical semivariogram ordinary Kriging type spatial interpolation technique to forecast the extent of~~ [28, 29]. Based on sampled clusters, we employed a spherical semivariogram ordinary Kriging-type spatial interpolation technique to forecast the extent of shared toilet facilities in Ethiopia for unsampled areas. The input for spatial prediction was the percentage of shared restrooms in each cluster. ~~In order to~~To determine the locations of

Formatted: Space Before: 0 pt, After: 0 pt

Formatted: Font: 14 pt

Formatted: Space Before: 6 pt, After: 0 pt

shared restroom clusters, Bernoulli-based model spatial scan statistics were applied. ~~[27].~~[30]. To suit the Bernoulli model, the houses without shared toilets were taken as controls, and the scanning window that moves over the research region with shared toilet facilities was taken as a case.

~~**RESULT**~~

RESULTS

Sociodemographic characteristics of the study participants

This study ~~includes 8,663~~included a total of 7,770 households~~ in total. Of~~. Among them, males ~~account~~constituted more than three-fourths (6,~~751 (~~046 or 77.~~93~~81%) of the household heads. ~~Of the survey ~~The majority of participants~~, the majority (69.47~~ (72.19%) lived in rural areas, and

Formatted: Font: Bold

Formatted: Space Before: 0 pt, After: 0 pt

Formatted: Font: 12 pt

Formatted: Space Before: 0 pt

Formatted: Normal (Web)

nearly half 4,128 (47.65(3,785 or 48.71%) of the household heads of home had no formal education.

As the age of household heads increased from 13-30 years to above 57 years, the magnitudeuse of shared toilet facilities -usage of the household decreased from 16.1720.35% to 6.18%9%, respectively. NearlyMore than two-fifths (39.95thirds (69.09%) of households found in metropolismetropolitan regions and about one-fourth (28.95%) of householdshousehold heads who is in urban residences (23.49%) used shared toilet facilities -**[Table 3].**

Table 3: Socio-demographic characteristics and shared toilet usage in Ethiopia, mini 2019 EDHS.

**Variables**	**Categories**	**Shared toilet facilities**		**Total weighted frequency (%)**
		**Yes (%) n = 819 (9.4610.5)**	**No (%) n = 7,844 (90.546,951 (89.5)**	
Age of household head (years)	13–3015–24	366 (16.17) 184 (20.35)	1899 (83.83722 (79.65)	2,266 (26.15906 (11.66)
	3125–40	188 (8.42370 (11.40)	2044 (91.58)2875 (88.60	2,232 (25.763245 (41.75)
	41–5654	139 (6.53136 (7.74)	1990(93.471616 (92.26)	2129 (24.581751 (22.53)
	>5755	126129 (6.1892)	19111739 (93.8208)	2036 (23.511868 (24.05)
Sex of household head	Male	501(7.42 (8.28)	6251(92.525545 (91.72)	6,751046 (77.9381)
	Female	318 (16.66)18.470	1,593 (83.341406 (81.53)	1,912724 (22.0719)
Educational attainment of household head	No education	211 (5.52228 (6.01)	3,876 (94.48556 (93.99)	4,120 (47.56)3,785 (48.71)
	Primary education	227(7.95244(8.79)	2,809530 (92.0521)	3069 2,775(35.4371
	Secondary & above	331 (23.60348 (28.71)	1,209 (76.40863 (71.29)	1,474 (17.01211 (15.58)

Formatted: Font: Bold

Formatted Table

Formatted: Font: 11 pt, Font color: Black

Formatted: Font: 11 pt, Font color: Black

Formatted: Font: 11 pt, Font color: Black

Formatted: Font: 11 pt, Font color: Black

Formatted: Font: 11 pt, Font color: Black

Formatted: Font: 11 pt, Font color: Black

Formatted: Font: 11 pt, Font color: Black

Formatted: Underline

Formatted: Font: 11 pt, Font color: Black

Formatted: Font: 11 pt, Font color: Black

Formatted: Font: 11 pt, Font color: Black

Formatted: Font: 11 pt, Font color: Black

Formatted: Font: 11 pt, Font color: Black

Formatted: Font: 11 pt, Font color: Black

Formatted: Font: 11 pt, Font color: Black

Formatted: Font: 11 pt, Font color: Black

Formatted: Font: 11 pt, Font color: Black

Formatted: Font: 11 pt, Font color: Black

Household family size	1-3	469 (16.5849 8 (17.96)	2359(83.422141 (82.04)	2,827 (32.64610 (33.59)
	4-6	249 (6.447.35)	3621(93.563140 (92.65)	3,870 (44.67889 (43.61)
	7 & above	101 (5.1672)	18641670 (94.8428)	1,966771 (22.6980)
Wealth index	Poor	60 (1.912.01)	3,073 (98.09) 2,929 (97.99)	3,133 (36.162,989 (38.47)
	Middle	36 (2.1835)	1,639516 (97.82) 65)	1,675553 (19.3498)
	Rich	723 (18.7522.39)	3,132 (81.252,506 (77.61)	3,855 (44.50229 (41.55)
**Community level variables**				
Residence	Urban	626 (23.49625 (28.95)	2038 (76.511,535 (71.05)	2,664 (30.75161 (27.81)
	Rural	193 (3.2345)	5,806415 (96.7755)	5,999 (69.25609 (72.19)
Region	Metropolis	173 (39.9569.09)	260 (60.0577 (30.91)	432 (4.99250 (3.22)
	Large centrals	530 (7.0168)	70396,373 (92.9932)	7,570 (87.386,904 (88.85)
	Small periphery	116 (17.5418.82)	545 (82.46500 (81.18)	661615 (7.6393)
Community poverty level	Low	651 (14.48711 (15.43)	3,850 (85.52898 (84.57)	4,502 (51.97610 (59.33)
	High	81 (1.92108 (3.41)	4,081 (98.083,052 (96.59)	4,161 (48.033,160 (40.67)

Formatted Table

Formatted: Font: 11 pt, Font color: Black
Formatted: Font: 11 pt, Font color: Black
Formatted: Font: 11 pt, Font color: Black
Formatted: Font: 11 pt, Font color: Black
Formatted: Font: 11 pt, Font color: Black
Formatted: Font: 11 pt, Font color: Black
Formatted: Font: 11 pt, Font color: Black
Formatted: Font: 11 pt, Font color: Black
Formatted: Font: 11 pt, Font color: Black
Formatted: Font: 11 pt, Font color: Black
Formatted: Font: 11 pt, Font color: Black
Formatted: Font: 11 pt, Font color: Black
Formatted: Font: 11 pt, Font color: Black
Formatted: Font: 11 pt, Font color: Black
Formatted: Font: 11 pt, Font color: Black
Formatted: Font: 11 pt, Font color: Black
Formatted: Font: 11 pt, Font color: Black
Formatted: Font: 11 pt, Font color: Black
Formatted: Font: 11 pt, Font color: Black
Formatted: Font: 11 pt, Font color: Black
Formatted: Font: 11 pt, Font color: Black
Formatted: Font: 11 pt, Font color: Black
Formatted: Font: 11 pt, Font color: Black
Formatted: Font: 11 pt, Font color: Black

The magnitude of shared ~~toilet~~sanitation facilities in Ethiopia

~~The~~ In the EDHS 2019, the magnitude of shared ~~toilet~~sanitation, facilities among households in Ethiopia ~~in EDHS 2019~~ was ~~9.46~~10.5% (95% CI: ~~8.86, 10.09) with the highest shared toilet facilities seen in Dire Dawa (44.6%) and the lowest (2.2%) seen in SNNP (Fig. 1).~~

~~**Types of shared toilet usage in Ethiopia**~~

~~Among households in Ethiopia that~~9.88, 11.24). Of these, more than two-thirds (69.5%), used shared ~~toilet facilities, four fifths 79.75% (95% CI: 77.56, 81.78) used~~ pit latrines with slabs ~~whereas 1.45% were used as~~, while only 0.6% used shared sanitation facilities connected to a flush ~~to~~ piped sewer system ~~type of sanitation facility (Fig. 2).~~. The highest prevalence of shared toilet facilities was observed in Addis Ababa (70.2%), whereas the lowest was in the Southern Nations, Nationalities, and Peoples' Region (2.4%) **(Fig. 1)**.

Factors associated with shared or limited access to improved sanitation service in Ethiopia

Since the models were nested, log-likelihood and deviance tests were performed for model comparison. The III-level binary logistic regression model was chosen because it had the largest LR (-~~2,669~~1438) and the smallest deviance test result (~~5,338~~2,876). The random effects of ICC, PCV, and MOR were evaluated.

The difference at the cluster level was responsible for almost ~~72~~79% of the variability in shared ~~toilet~~sanitation facilities among sample families, according to the ICC in the null model. A household from a cluster with a high risk of domestic violence had ~~15.54~~28.75 times higher odds of having shared toilet facilities than a household from a cluster with a lower risk of shared toilet facilities, according to the MOR value in the null model (~~15.54~~28.75), on the other hand, if you randomly selected two households from two different clusters. This demonstrated the considerable variation in the use of communal restrooms among various populations.

Formatted: Pattern: Clear
Formatted: Pattern: Clear
Formatted: Pattern: Clear
Formatted: Pattern: Clear
Formatted: Pattern: Clear
Formatted: Pattern: Clear
Formatted: Pattern: Clear
Formatted: Pattern: Clear
Formatted: Pattern: Clear (White)

Additionally, the PCV valve in the final model demonstrated that characteristics at both the individual and community levels simultaneously explained nearly ~~80~~76% of the variation in shared toilet facilities among study families**[Table 4].**

~~According to~~Based on the ~~chosen model~~results from Model III~~ result,~~, there was a positive association between improved shared ~~toilet~~sanitation facilities ~~are significantly correlated~~and several factors such as individuals with ~~individual level variables like the household head's age,~~ higher educational attainment~~, size of the~~ and from greater household~~, and~~ wealth~~ status, while community level variables like residence, region, and community level poverty are significantly correlated with it.~~, those living in urban areas and metropolitan regions.

As the age of household heads increased to ~~31~~15–24 years, 25–40~~, 41–56~~ years, and ~~≥57~~over 55 years, the odds of using shared toilet facilities decreased by ~~30%, 47%, and~~ 33%, 46%, and 52%, respectively [AOR~~=~~ = 0.~~70~~66; 95% CI~~;~~: 0.~~58~~50, 0.~~86~~89], [AOR~~=~~ = 0.~~53~~54; 95% CI~~;~~: 0.~~42~~37, 0.~~66~~79], and [AOR~~=~~ = 0.~~54~~48; 95% CI~~;~~: 0.~~42~~33, 0.~~69~~71]. The odds of using shared toilet facilities were doubled among household heads ~~who have~~with above-primary ~~educational status were increased by 31%~~education [AOR = ~~1.31~~2.43; 95% CI: 1.80, 3.28].

Households with a middle or rich wealth index were 2 and 6 times more likely to use shared toilet facilities compared to poor households [AOR = 2.32; 95% CI: 1.~~04, 1.64].~~

~~As the family members of the household increase to 4-6 and above six members, the odds of using shared toilet facilities decrease by~~ 35~~% and 45% [AOR = 0.65; 95% CI = 0.54, 0.77~~, 3.96] and [AOR = ~~0.55~~6.23; 95% CI~~ = 0.42, 0.73~~: 3.84, 10.11], respectively. ~~Households with a rich wealth index were 4.88~~Individuals living in urban areas were 7.6 times more likely to use shared toilet facilities compared to those in rural areas [AOR = 7.60; 95% CI: 3.47, 16.67]. Residents of metropolitan regions were 26 times more likely, and those in small periphery regions were 5 times more likely to use shared toilet facilities ~~as compared to poor households [AOR = 4.88; 95% CI:~~

Formatted: Font color: Text 1
Formatted: Font color: Text 1
Formatted: Normal
Formatted: Font color: Text 1
Formatted: Font color: Text 1
Formatted: Font color: Text 1
Formatted: Font color: Text 1
Formatted: Font color: Text 1
Formatted: Font color: Text 1
Formatted: Font color: Text 1

3.38, 7.06]. On the other hand, households that live in high levels of community poverty were 1.73 times higher than those at low levels of community poverty [AOR = 1.73; 95% CI: 1.04, 2.86].

People who live in rural households were 92% less likely to use shared toilet facilities as compared to those in urban areas large central regions [AOR = 25.83; 95% CI: 10.1, 66.3] and [AOR = 0.085.01; 95% CI: 0.05, 0.14]. Living in the large central regions was 83% less likely to use shared toilet facilities as compared to metropolitan cities [AOR = 0.17; 95% CI: 0.12, 0.26] 2.40, 10.48], respectively[**Table 4**].

Formatted: Font: Not Bold

Formatted: Font: Not Bold

Formatted: Space Before: 0 pt, After: 0 pt

Formatted: Font: Not Bold

Formatted: Normal, Line spacing: Double

Table 4: Multilevel analysis of factors associated with shared toilet facilities usage among households in Ethiopia.

Formatted Table

**Variables**	**Categories**	**Null model**	**Model I AOR [95% CI]**	**Model II AOR [95% CI]**	**Model III AOR [95% CI]**
Age of household head (years)	13–3015–24		1.00	-----------	1.00
	3125–40		0.7467 [0.6050, 0.91]*	-----------	**0.7066 [0.5850, 0.86]**89]***
	41–5654		0.56 [0.4438, 0.69]***81]**	-----------	**0.5354 [0.4237, 0.66]**79]***
	>5755		**0.5849 [0.4534, 0.7372]*****	-----------	**0.5448 [0.4233, 0.6971]*****
Sex of household head	Male		1.00	-----------	1.00
	Female		1.09[17 [0.86, 1.90, 1.3237]	-----------	1.0223 [0.8492, 1.2347]
Educational attainment	No education		1.00	-----------	1.00
	Primary education		1.1636 [0.9499,1.4578]	-----------	1.0831 [0.87,98, 1.3473]

of household head	Secondary& above		12.54 [1.22, 1.9388, 3.44]**	------------	2.43 [1.31 [1.04, 1.6480, 3.28]*
Household family size	1-3		1.00	------------	1.00
	4-6		0.6175 [0.51, 0.73]*61, 1.03]	------------	0.6576 [0[illegible] 0.77]***60, 1.01]
	7 & above		0.4988 [0.37, 0.65]**62, 1.26]	------------	0.55 [0.42, 0.73]*[illegible] [68, 1.38]
Wealth index	Poor		1.00	------------	1.00
	Middle		2.66 [1.24 [0.79, 1.98]55, 4.56]**	------------	2.32 [1.18 [0[illegible] 1.88]35, 3.96]*
	Rich		8.08 [5.71, 11.4510. 25 [6.39, 16.41]**	------------	4.886.23 [3[illegible] 7.0684, 10.11]***
**Community level variables**					
Residence	Urban		------------	14.6 [6.61, 32.21] ***1.00	7.60 [3[illegible] 16.67]***1.00
	Rural		------------	1.000.05 [0.03, 0.08]***	1.000.08 [0[illegible] 0.14]***
Region	Metropolis		------------	1.0028.97 [11.21, 74.87]***	1.0025.83 [10.1, 6[illegible] ***
	Large centralSmall periphery		------------	0.18 [0.11, 0.28]*4.51 [2.14, 9.49]***	0.17[0.12, 0.26]*[illegible] [2.40, 10.48] ***
	Small peripheryLarge central		------------	0.77 [0.45, 1.31]**00	0.81[0.47, 1.37]00[illegible]
Community poverty usage	Low		------------	1.00	1.00
	High		------------	1.01 [0.65, 1.5823 [0.11, 0.49]	0.65 [0.29, 1.73 [1[illegible] 2.86]*39]
**Random effect**					
	Variance	12.5 [8.3499, 17.46]	6.75 [4.4984, 9.41]	1.733.27 (2.34, 4.55)	1.653.05 [2.17, 4.29]
	ICC	0.7279 [0.73, 0.84]	0.5867 [0.59, 0.74]	0.3450 [0.42, 0.58]	0.3348 [0.40, 0.57]
	MOR	15.5428.75	7.4611.80	3.485.57	3.395.25
	PCV	Reff	0.46	0.7974	0.8076
**Model comparison**					
	Log-likelihood	-3,0321654	-28321515	-2,7941544	-2,6691438
	Deviance	6,0643.308	5,6643,030	5,5883,088	5,3382,876
	Mean VIF	---	1.59	2.18	1.96

** = P-value < 0.05, ** = Pvalue < 0.01, *** = Pvalue < 0.001*

ICC = Inter cluster correlation coefficient, MOR =Median odds ratio, PCV= proportional change in variance. AOR=adjusted odds ratio; CI= confidence intervale, VIF=Variance Inflation Factors

Formatted: Font: Not Bold
Formatted: Font: Not Bold
Formatted: Font: Not Bold
Formatted: Font: Not Bold
Formatted: Font: Not Bold
Formatted: Font: Not Bold
Formatted: Font: Not Bold
Formatted: Font: Not Bold
Formatted: Font: Not Bold
Formatted: Font: Not Bold
Formatted: Font: Not Bold
Formatted: Font: Bold
Formatted: Font: Bold
Formatted: Font: Bold
Formatted: Font: Not Bold
Formatted: Font: Not Bold
Formatted: Font: Not Bold
Formatted: Font: Not Bold

Spatial analysis of shared toilet facilities ~~utilisation~~ utilization among households in Ethiopia

Spatial autocorrelation analysis of shared toilet facilities

Ethiopia's shared restroom spatial autocorrelation data revealed a strong positive spatial autocorrelation throughout the nation's regions. It was discovered to be grouped with the value of the Global Moran's Index: 1.~~393~~78 with (p< 0.001) (**Fig. ~~3~~2**).

Hotspot ~~analysis~~Analysis of shared toilet facilities among household in Ethiopia

The Getis-Ord Gi* hotspot analysis showed that shared toilet facilities were more practised in Addis Ababa, and Dirrie Dawa, whereas the SNNPR (South Nations Nationalities and People's Region), and Beneshangul Gumuz regions were the cold spot areas (**Fig. ~~4~~3**).

Significant windows and SaTscan analysis of shared toilet facilities among households in Ethiopia

The ~~SaTscan~~SaTScan analysis of shared toilet facilities among households in Ethiopia ~~showed that~~identified 86 primary clusters and 29 secondary ~~were detected for having shared toilet facilities.~~clusters. The primary clusters were centered at coordinates 8.771915 N, 40.335915 E, with a radius of 206.97 km~~ radius,~~, and were located in Addis Ababa, ~~Northern~~northern parts of Oromia, southern parts of Amhara and Afar, and ~~Dirrie~~Dire Dawa. Households ~~who were found in the~~within these primary ~~window~~clusters were 5 times more likely to use shared toilet facilities ~~care than out in window regions~~compared to those outside these clusters (RR = 5.0, P-value~~<~~ < 0.001) (**Table 5, Fig.~~5~~4).**

Table 5: Significant spatial clusters of shared toilet facilities among households in Ethiopia using 2019 mini-EDHS~~.~~.

Clusters	Enumeration areas (clusters) detected	Coordinate/radius	Population	Cases	RR	LLR	P-valu

Formatted: Space After: 0 pt

Formatted: Space After: 0 pt

Formatted: Pattern: Clear

Formatted: Pattern: Clear

Formatted: Font: Times New Roman

Formatted: Font: Times New Roman

Formatted: Font: Times New Roman

Formatted: Font: Times New Roman

Formatted: Font: Times New Roman

Formatted: Font: Times New Roman

Formatted: Font: Times New Roman

Formatted: Font: Times New Roman

Formatted: Font: Times New Roman

1ry (86)	105, 88, 28, 41, 102, 106, 42, 127, 40, 104, 69, 90, 101, 43, 103, 108, 272, 269, 268, 271, 110, 280, 278, 273, 279, 270, 267, 264, 266, 277, 305, 275, 276, 265, 263, 256, 274, 304, 258, 257, 261, 260, 262, 50, 259, 303, 281, 296, 287, 68, 282, 302, 286, 284, 288, 283, 111, 294, 285, 291, 292, 293, 295, 297, 290, 289, 175, 231, 233, 246, 244, 232, 243, 237, 234, 236, 301, 298, 235, 242, 247, 241, 240, 253, 238, 239	8.771915 N, 40.335915 E / 206.97 km	2447	851	5.04	181.4-489.78	<0.001
2nd (29)	281, 282, 284, 283, 287, 285, 286, 288, 296, 291, 297, 292, 294, 290 , 289, 295, 302, 264, 273, 267, 263, 270, 276, 265, 275, 266, 258, 271,	10.589922 N, 34.352539 E / 88.60 km	505	245	2.28	7.0-181.3	<0.001

Kiringing interpolation of shared toilet facilities among households in Ethiopia

The Kriging interpolation ~~methods of~~method for shared toilet facilities among households in Ethiopia ~~showed~~indicated that high-risk areas, such as Addis Ababa and Dire Dawa, had predicted ~~shared toilet facilities~~usage rates ranging from 50% to 61~~% and are in Addis Ababa and Dire Dawa. The lower~~%. Conversely, the lowest predicted ~~area was seen in~~usage rates were observed in the SNNPR, Gambela, Beneshangul Gumuz, and Oromia regions, ranging from 0% to 12% (**Fig.** ~~6).~~5)

Formatted: Font: Times New Roman
Formatted: Font: Times New Roman
Formatted: Font: Times New Roman
Formatted: Font: Times New Roman
Formatted: Font: Times New Roman
Formatted: Font: Times New Roman
Formatted: Font: Times New Roman
Formatted: Font: Times New Roman
Formatted: Font: Times New Roman
Formatted: Font: Times New Roman
Formatted: Font: Times New Roman
Formatted: Font: Times New Roman
Formatted: Font: Times New Roman
Formatted: Font: Times New Roman
Formatted: Font: Times New Roman
Formatted: Font: Times New Roman
Formatted: Space Before: 0 pt, After: 0 pt
Formatted: Space Before: 0 pt, After: 0 pt
Formatted: Font: Bold
Formatted: Font: 12 pt
Formatted: Normal (Web), Line spacing: Double

DISCUSSION

Formatted: Font: Bold

This study was conducted to assess the magnitude of shared toilet facilities and their determinants among households in Ethiopia. Based on this, the prevalence of shared toilet facilities in Ethiopia was 9.46% (95% CI: 8.86, 10.09). This is higher than a study by the WHO and UNICEF Joint Monitoring Program in Ethiopia (7%).

[3]. It is also higher than Yemen (4%), and Eritrea (5%). But it is lower than studies in Kenya (21%), Ghana (57%), and Uganda (14%) [3]. This variation might be due to the involvement of different community initiative programs that have a better approach toward the reduction of unimproved sanitation practices and the achievement of the desired sanitation program [16, 28].

In this study, as the age of household heads increases, the shared toilet facilities of households decrease. This is in line with a study done in India that shows unimproved sanitation practice decreases among the oldest household members [29]. The reason might reflect that, as age increases, disability or incontinence might occur, which makes it difficult to defecate outside the home [29]. The other just might be older people, on average, who are unable to move more freely

outside their homes and enact their preferences. Furthermore, elderly people may perceive they have a poorer immune system to many diseases caused by unimproved sanitation.

In this study, as the educational status of household heads increased, the odds of using shared toilet facilities also increased. This is because one-third (32.23%) of households in Ethiopia use open defecation[14] and those who have at least formal education use shared toilet facilities, which has much less serious consequences for health, safety, and dignity than open defecation [2]. Moreover, educated household heads can understand the effects of open defecation and the relevance of having sanitation facilities even if it is limited service [30, 31]. According to the World Health Organization, education about the relationship between sanitation, hygiene, and health raises community demand for better sanitation facilities [32]. According to previous research, a high degree of education increases awareness and positive attitudes toward selecting comparatively more upgraded sanitation facilities [33]. Specifically, educated women prefer safe and sanitary facilities, maintain privacy and are of reasonable quality during their menstrual cycle. Moreover, well-educated households with higher incomes had greater access to upgraded sanitary facilities [34, 35]. Education will help to reduce the health dangers associated with open defecation.

This study's findings showed that as the number of family members in the household increases, the odds of using shared toilet facilities decrease. This is supported by studies that showed that households with large sizes are more likely to defecate in the open than those with smaller sizes [35, 36]. This suggests that heads of large households may be burdened with the need to cater to basic needs [37]. If there are large numbers of household members, people cannot tolerate keeping the order to use the shared toilet and might prefer to use open defecation.

Larger households may have a greater opportunity to build a latrine because certain members may be employed and earning money. Shared toilet facilities suffer from "the tragedy of the commons,"

~~as illustrated by Hardin [38], in which no one is willing to make personal sacrifices for the benefit of all consumers. Small family households may find it difficult to exclude large family household users who benefit from shared facilities, but misuse and a lack of cleaning diminish the ability of other users to use the facility. The number of users has an impact on the hygiene of shared toilet facilities because a large number of users makes it harder to coordinate, make collective choices, and apply rules[39].~~

This study was conducted to assess the magnitude of improved shared toilet facilities and their determinants among households in Ethiopia. Based on this, the prevalence of improved shared toilet facilities in Ethiopia was 10.5% (95% CI: 9.88, 11.24). This is higher than a study by the WHO and UNICEF Joint Monitoring Program in Ethiopia (7%) [7]. It is also higher than in Yemen (4%) and Eritrea (5%), but lower than the global estimate reported in meta-analyses (67%) [4], in Kenya (21%), Ghana (57%), and Uganda (14%) [7]. This variation may be attributed to the different community initiative programs that employ more effective approaches to reducing unimproved sanitation practices and achieving desired sanitation outcomes [18, 31].

In this study, as the age of household heads increases, the usage of shared toilet facilities among households decreases. This is consistent with a study conducted in India, which found that unimproved sanitation practices decrease among older household members [32]. This trend may reflect that as individuals age, they may experience disabilities or incontinence, making it more challenging to use outdoor or shared sanitation facilities [32]. Another factor could be that older individuals, on average, may have reduced mobility and greater difficulty moving freely outside their homes to access sanitation facilities. Additionally, elderly people might be more concerned about their weakened immune systems and the increased risk of diseases associated with unimproved sanitation

In this study, as the educational status of household heads increased, the odds of using shared toilet facilities also increased. This is because one-third (32.23%) of households in Ethiopia use open defecation[3] which is more prevalent in less socioeconomic regions. Individuals with at least a formal education are more likely to use shared toilet facilities, which generally have fewer severe consequences for health, safety, and dignity compared to open defecation [1]. Furthermore, educated household heads are more likely to recognize the importance of having sanitation facilities, even if they are limited or shared [33, 34]. According to the World Health Organization report, education can raise the community's demand for better sanitation facilities [35]. Moreover, a higher level of education enhances awareness and fosters positive attitudes toward choosing relatively more upgraded sanitation facilities [36]. Specifically, educated women are more likely to prefer safe and sanitary facilities that offer privacy and maintain good quality during their menstrual cycle [37]. Moreover, well-educated households with higher incomes had better access to upgraded sanitation facilities [38].

Households that have a rich wealth index are more likely to use the shared toilet as compared to poor households. This is in line with Ethiopia [4], Nigeria ~~[40]~~[39], and Gahanna ~~[37].~~[40]. This might be because the majority of shared toilet facilities ~~-~~are found in urban areas with a good income-earning population group as compared to open defecation practices that have been taking place in rural areas of low-income countries ~~[41].~~[41]. In addition, poor households may not have enough place and capacity to construct private and shared toilets and their only option is open defecation. Furthermore, poor ~~urban~~ households found in developing countries are mainly housed in slums that lack essential infrastructure ~~[35]. This study suggests that sanitation is an investment; peri-urban inhabitants took the effort to get improved toilet facilities because they had consistent income to support their planning.~~[37].

~~This study finding also showed that peoples who live in rural households were less likely to use shared toilet facilities than urban households. This is consistent with the study, where the use of~~

Formatted: Font: 12 pt
Formatted: Font: 12 pt
Formatted: Font: 12 pt
Formatted: Font: 12 pt
Formatted: Font: 12 pt
Formatted: Font: 12 pt
Formatted: Font: 12 pt
Formatted: Font: 12 pt
Formatted: Font: 12 pt

shared toilet facilities was less common in rural areas and open defecation is a common practice (37%) in rural areas of Ethiopia [42]. This could be because many governments do not prioritize rural sanitation on their national agendas and do not make progressive budgetary support as well as essential legislative and institutional reforms [43]. Besides, the previous study finding suggested that rural households' willingness to pay for 'improved' latrines is minimal [44]. Moreover, rural households are uneducated, and they believe that open defecation is a routine sanitation service [31].

The study also found that individuals living in rural households were less likely to use improved shared toilet facilities compared to those in urban areas. This finding is consistent with other research indicating that shared toilet facilities are less common in rural areas, where open defecation remains prevalent, affecting 37% of the rural population in Ethiopia [42]. This may be because many governments do not prioritize rural sanitation in their national agendas, often lacking progressive budgetary support as well as essential legislative and institutional reforms [43]. Besides, the previous study findings suggested that rural households' willingness to pay for 'improved' latrines is minimal [44]. Moreover, rural households are uneducated, and they believe that open defecation is a routine sanitation service [34]. On the other side, sanitation is an investment, and peri-urban inhabitants made the effort to get improved toilet facilities because they had relatively consistent income to support their planning.

The other finding showed that households who live in large central regions were less likely to use shared toilet facilities as compared to metropolitan cities. The spatial analysis result also showed that shared toilet facilities were more commonly practiced in Addis Ababa and DirrieDire Dawa, whereas the SNNPR (South Nations Nationalities and People's Region) and Beneshangul Gumuz regions were the cold spots. This is in line with a study in Mozambique that shows that shared toilet facilities are increasingly common in low-income countries in rapidly growing urban areas[2][1]. Moreover, in Ethiopia, as a result of internal conflict and drought, informal settlements

Formatted: Space Before: 0 pt, After: 0 pt

and refugee camps that use shared toilet facilities are more common in ~~metropolite~~metropolitan cities. The other possible explanation is that those large Centrals contained rural households that practiced open defecation rather than shared and improved sanitation.

The strengths of this study lie in the use of nationally representative, high-quality standardized data, which allows for generalization at the country level. However, a limitation of the study is that being cross-sectional, it cannot establish cause-and-effect relationships.

Conclusion and recommendation

Formatted: Normal (Web), Line spacing: Double

The prevalence of improved shared ~~toilet facility~~sanitation services usage among households in Ethiopia is relatively low. Age, educational attainment of the household head, ~~household family size,~~ wealth status, residence, and ~~the~~ region were found to be ~~significant~~significantly associated factors ~~in~~ with improved shared ~~toilet~~sanitation facilities in Ethiopia. ~~Even though the prevalence of~~Moreover, there was a non-random spatial distribution of improved shared ~~toilet facilities is low, the~~sanitation services in Ethiopia mainly found in Addis Ababa and Dire Dawa. The government

of Ethiopia and other stakeholders should provide economic support for those poorto low-income households that facilitateand rural residents to construct improved shared sanitation use. Furthermore, thefacilities. Additionally, policy in Ethiopia should emphasize user awareness and practices through financial and education. Because, if properly managed, household shared toilet literacy to enhance access to and usage of improved shared sanitation facilities in informal communities can be considered improved sanitation.

Strength and Limitation of the study

High response rates, national coverage, high-quality interviewer training, standardized data collection procedures across countries, and consistent content over time, whereas using cross sectional study design which can not predict cause-effect issue, particularly in rural residences and large central regions. Further qualitative studies are needed to explore the behavioral and socio-cultural factors that may be the limitation of the study.prevent individuals from utilizing improved shared sanitation facilities.

Abbreviations

AOR: Adjusted Odds Ratio, CI: Confidence Interval, CSA: Central Statistical Agency, EDHS: Ethiopian Demographic and Health Survey, HR: Household Record, MOH: Ministry Of Health, OD, Open Defecation, SDG: Sustainable Development Goal.

Declarations

Ethical ~~approval~~Approval and Consent to ~~participate~~Participate

The study doesn't involve the collection of information from subjects, secondary data analysis was done. Ethical approval and consent to participate are not applicable. Since the study is a secondary data analysis based on DHS data.

Consent for publication

Not applicable

Availability of data and materials

Data were accessed after registration, submission of the request, and permission granted via the link *https://dhsprogram.com/data/new-user-registration.cfm*.

The shapefile data was created by GADM, found here: https://gadm.org/index.html, and was created under this license.

Competing Interests

The authors declare that they have no competing interests.

Formatted: Font: Bold

Formatted: Line spacing: 1.5 lines

Formatted: Space After: 0 pt

There are no financial, non-financial, or commercial organizations competing for interests

Funding

No funding was obtained for this study.

Authors' contribution

The conception of the work, design of the work, acquisition of data, analysis, and interpretation of data ~~was~~were done by DGB, and BTA. Data curation, drafting the article, revising it critically for intellectual content, validation, and final approval of the version to be published was done by BTA and DGB. Both authors read and approved the final manuscript.

~~**Acknowledgements**~~

Acknowledgments

I would like to thank the "measure DHS program" for providing the data set.

Figure legends

Fig. 1: Magnitude of shared toilet facilities among households in Ethiopia, using the 2019 EDHS.

~~**Fig. 2:**Types of shared toilet facilities among households in Ethiopia, using the 2019 EDHS.~~

~~**Fig.3**~~**Fig.2**: Spatial autocorrelation analysis of shared toilet facilities among households in Ethiopia using 2019 mini-EDHS. The base map for the shapefile was sourced from: https://gadm.org/

Fig.~~**4**~~**3**: Hotspot analysis of shared toilet facilities among households in Ethiopia using 2019 mini-EDHS. The base map for the shapefile was sourced from: https://gadm.org/ .

Fig. ~~**5**~~**4**: SaTscan analysis of shared toilet facilities among households in Ethiopia using 2019 mini-EDHS.: The base map for the shapefile was sourced from: https://gadm.org/

Fig. ~~**6**~~**5**: Kiringing interpolation of shared toilet facilities among households in Ethiopia using 2019 mini-EDHS.:The base map for the shapefile was sourced from: https://gadm.org/.

Formatted: Font: 16 pt, Bold, English (United States)

Formatted: Space Before: 0 pt

Formatted: Indent: Left: 0"

References

~~1.~~ ~~Organization, W.H.~~1. Shiras, T., ~~and UNICEF, *Progress on Drinking Water, Sanitation and Hygiene Update and SDG Baselines 2017*. 2017, WHO, Geneva.~~

~~2.~~ ~~Shiras, T.,~~ et al., *Shared Sanitation Management and the Role of Social Capital: Findings from an Urban Sanitation Intervention in Maputo, Mozambique.* Int J Environ Res Public Health, 2018. **15**(10).

~~3.~~ 2. World Health Organization and UNICEF, *Progress on Drinking Water, Sanitation and Hygiene Update and SDG Baselines 2017*. 2017, WHO, Geneva.

3. Central Statistical Agency (CSA) [Ethiopia] and ICF. 2016, *Ethiopia Demographic and Health Survey 2016*. Addis Ababa, Ethiopia, and Rockville, Maryland, USA: CSA and ICF.

~~, W.H., *Progress on drinking water,*~~4. Sprouse, L., et al., *Shared* sanitation ~~*and hygiene: 2017 update and SDG baselines*. 2017~~*in informal settlements: A systematic review and meta-analysis of prevalence, preferences, and quality*. International Journal of Hygiene and Environmental Health, 2024. **260**: p. 114392.

~~4~~5. *Simiyu, S., Why shared toilets in informal settlements may pose a serious health risk, available on https://theconversation.com/why-shared-toilets-in-informal-settlements-may-pose-a-serious-health-risk-94339.* 2018.

6. Abebe, T.A. and G.T. Tucho, *Open defecation-free slippage and its associated factors in Ethiopia: a systematic review.* Syst Rev, 2020. **9**(1): p. 252.

~~5~~7. World Health Organization, *Progress on drinking water, sanitation and hygiene: 2017 update and SDG baselines.* 2017.

8. Evans, B., et al., *Limited services? The role of shared sanitation in the 2030 agenda for sustainable development*. 2017, IWA Publishing.

~~6~~9. Spears, D., A. Ghosh, and O. Cumming, *Open defecation and childhood stunting in India: an ecological analysis of new data from 112 districts.* PloS ~~one~~One, 2013. **8**(9): p. e73784.

~~7~~10. John, J.R., et al., *Determinants of early initiation of breastfeeding in Ethiopia: a population-based study using the 2016 demographic and health survey data.* BMC Pregnancy Childbirth, 2019. **19**(1): p. 69.

~~8~~11. Debela, T.H., et al., *Fecal contamination of soil and water in sub-Saharan Africa cities: The case of Addis Ababa, Ethiopia.* Ecohydrology & Hydrobiology, 2018. **18**(2): p. 225-230.

~~9~~12. Gebru, T., M. Taha, and W. Kassahun, *Risk factors of diarrhoeal disease in under-five children among health extension model and non-model families in Sheko district rural community, Southwest Ethiopia: a comparative cross-sectional study.* BMC ~~public health~~Public Health, 2014. **14**(1): p. 1-6.

~~10~~13. Liu, L., et al., *Global, regional, and national causes of child mortality: an updated systematic analysis for 2010 with time trends since 2000.* The ~~lancet~~Lancet, 2012. **379**(9832): p. 2151-2161.

~~11~~14. UNICEF, *Sanitation issue*. Addis Ababa: Ethiopian Newsletter, 2004.

12. nations, U., 15. United *nations*Nations, *United Nations Department of Economic and Social Affairs, Transformational benefits' of ending outdoor defecation: Why toilets matter avilable on ,https://www.un.org/development/desa/en/news/sustainable/world-toilet-day2019.html.available on,https://www.un.org/development/desa/en/news/sustainable/world-toilet-day2019.html.* 2018.

13. Simiyu, S., *Why shared toilets in informal settlements may pose a serious health risk, avaliable on https://theconversation.com/why-shared-toilets-in-informal-settlements-may-pose-a-serious-health-risk-94339*. 2018.

1416. World Health Organization, *Progress on drinking water, sanitation, and hygiene: 2017 update and SDG baselines. Available at: https://iris.who.int/bitstream/handle/10665/258617/9789241512893eng.pdf?sequence=1.* 2017.

17. Central Statistical Agency (CSA) [Ethiopia] and ICF. 2016, *Ethiopia Demographic and Health Survey 2016*. Addis Ababa, Ethiopia, and Rockville, Maryland, USA: CSA and ICF.

15. Research, B., *Outcome evaluation of Community-Led Total Sanitation and Hygiene (CLTSH) Program in Ethiopia from 2012-2015*. 2016, UNICEF Addis Ababa.

1618. Kar, K. and R. Chambers, *Handbook on community-led total sanitation.* 2008.

1719. Leshargie, C.T., et al., *Household latrine utilization and its association with educational status of household heads in Ethiopia: a systematic review and meta-analysis.* BMC Public Health, 2018. **18**(1): p. 1-12.

1820. Corsi, D.J., et al., *Demographic and health surveys: a profile.* International journal of epidemiology, 2012. **41**(6): p. 1602-1613.

Formatted: Font: Not Italic

~~19~~21. Worldometer, *African Countries by Population (~~2021~~2024) - Worldometer ~~https://www.worldometers.info/population/countries-in-africa-by-population/~~https://www.worldometers.info/population/countries-in-africa-by-population/*. ~~2021~~2024.

~~20~~22. Ethiopian Public Health Institute, a.I., *Ethiopia mini demographic and health survey 2019: key indicators. J Chem Information Model. 53:1689–1699.* 2019.

23. Croft, T., M. Aileen, and C. Marshall, *Allen,(2018).* Guide to DHS statistics.

~~21~~24. Teshale, A.B. and G.A. Tesema, *Magnitude and associated factors of unintended pregnancy in Ethiopia: a multilevel analysis using 2016 EDHS data.* BMC pregnancy and childbirth, 2020. **20**(1): p. 329-329.

~~22~~25. Liyew, A.M. and A.B. Teshale, *Individual and community level factors associated with anemia among lactating mothers in Ethiopia using data from Ethiopian demographic and health survey, 2016; a multilevel analysis.* BMC Public Health, 2020. **20**: p. 1-11.

~~23~~26. Merlo, J., et al., *A brief conceptual tutorial of multilevel analysis in social epidemiology: linking the statistical concept of clustering to the idea of contextual phenomenon.* Journal of Epidemiology & Community Health, 2005. **59**(6): p. 443-449.

~~24~~27. Merlo, J., et al., *A brief conceptual tutorial on multilevel analysis in social epidemiology: interpreting ~~neighbourhood~~neighborhood differences and the effect of ~~neighbourhood~~neighborhood characteristics on individual health.* Journal of Epidemiology & Community Health, 2005. **59**(12): p. 1022-1029.

~~25~~28. McMillen, D.P., *Geographically weighted regression: the analysis of spatially varying relationships*. 2004, Oxford University Press.

~~26~~29. exchange, S., *Choosing the value of Moran's I to say the existence of spatial correlation; access date: 22 ~~march~~March 2023. available at:*
~~https://gis.stackexchange.com/questions/269013/choosing-value-of-morans-i-to-say-~~

Formatted: Left

~~existence-of-spatial-correlation.~~https://gis.stackexchange.com/questions/269013/choosing-value-of-morans-i-to-say existence-of-spatial-correlation. 2018.

~~27~~30. Kulldorff, M., *A spatial scan statistic.* Communications in Statistics-Theory and methods, 1997. **26**(6): p. 1481-1496.

~~28~~31. Abebe, T.A. and G.T. Tucho, *Open defecation-free slippage and its associated factors in Ethiopia: a systematic review.* Systematic reviews, 2020. **9**(1): p. 1-15.

~~29~~32. Coffey, D., et al., *Revealed preference for open defecation.* Economic & Political Weekly, 2014. **49**(38): p. 43.

~~30~~33. Paavola, J., *Livelihoods, vulnerability and adaptation to climate change in Morogoro, Tanzania.* Environmental Science & Policy, 2008. **11**(7): p. 642-654.

~~31~~34. Tumwine, J., et al., *Sanitation and hygiene in urban and rural households in East Africa.* International journal of environmental health research, 2003. **13**(2): p. 107-115.

~~32~~35. Project, T.B., *EDUCATION AND IMPROVING SANITATION IN DEVELOPING COUNTRIES.* ~~https://borgenproject.org~~https://borgenproject.org › SEPTEMBER 26, 2016.

~~33~~36. Wei, F. and V.K. Pillai, *Sanitation in India: Role of women's education.* 2014.

~~34. Nyambe, S., L. Agestika, and T. Yamauchi, *The improved and the unimproved: Factors influencing sanitation and diarrhoea in a peri-urban settlement of Lusaka, Zambia.* PloS one, 2020. **15**(5): p. e0232763.~~

~~35~~37. Donacho, D.O., G.T. Tucho, and A.B. Hailu, *Households' access to safely managed sanitation facility and its determinant factors in Jimma town, Ethiopia.* Journal of Water, Sanitation and Hygiene for Development, 2022. **12**(2): p. 217-226.

38. Nyambe, S., L. Agestika, and T. Yamauchi, *The improved and the unimproved: factors influencing sanitation and diarrhea in a peri-urban settlement of Lusaka, Zambia.* PloS one, 2020. **15**(5): p. e0232763.

39. Abubakar, I.R., *Exploring the determinants of open defecation in Nigeria using demographic and health survey data.* Science of the total environment, 2018. **637**: p. 1455-1465.

~~36. Dagaga, D.T. and G.D. Geleta, *Water and Latrine Services and Associated Factors among Residents of Negele Town, Southeast Ethiopia: A Cross-Sectional Study.* Journal of Environmental and Public Health, 2022. **2022**.~~

~~37~~40. Osumanu, I.K., E.A. Kosoe, and F. Ategeeng, *Determinants of open defecation in the Wa municipality of Ghana: empirical findings highlighting sociocultural and economic dynamics among households.* Journal of environmental and public health, 2019. **2019**.

~~38. Hardin, G., *The tragedy of the commons: the population problem has no technical solution; it requires a fundamental extension in morality.* science, 1968. **162**(3859): p. 1243-1248.~~

~~39. Simiyu, S., et al., *Determinants of quality of shared sanitation facilities in informal settlements: case study of Kisumu, Kenya.* BMC public health, 2017. **17**(1): p. 1-13.~~

~~40. Abubakar, I.R., *Exploring the determinants of open defecation in Nigeria using demographic and health survey data.* Science of the total environment, 2018. **637**: p. 1455-1465.~~

41. Galan, D.I., S.-S. Kim, and J.P. Graham, *Exploring changes in open defecation prevalence in sub-Saharan Africa based on national level indices.* BMC ~~public health~~Public Health, 2013. **13**(1): p. 1-12.

42. Beyene, A., et al., *Current state and trends of access to sanitation in Ethiopia and the need to revise indicators to monitor progress in the Post-2015 era.* BMC ~~public health~~Public Health, 2015. **15**(1): p. 1-8.

43. ~~Blogs, W.B.,~~World Bank, *Why rural sanitation matters.* ~~https://blogs.worldbank.org,~~https://blogs.worldbank.org, NOVEMBER 14, 2019.

44. Affairs, M.o.F., *Rural Water and Sanitation.* ~~https://www.oecd.org~~https://www.oecd.org Number 6 · July 2012.

Formatted: Tab stops: 1.02", Left

Date: September 1, 2024

Alison Parker

Academic Editor of Plos One Journal

Re: ***Spatial distribution and determinants of improved shared toilet facilities among households in Ethiopia: Using 2019 mini-Ethiopian Demographic and Health Survey (Submission ID:*** **PONE-D-24-20290*)***

Dear Editor,

We are grateful for the opportunity to revise our manuscript for further consideration for publication in Plos One Journal.

We have addressed the reviewer's comments and suggestions. Our point-by-point response describes all changes in the manuscript text. We have indicated the changes in track changes in the revised manuscript. We hope that you will find the revised manuscript acceptable for publication.

Yours sincerely,

Baye Tsegaye Amlak

Corresponding Author

A point-by-point response to the reviewer's comment

We thank the editor and reviewer for their time and effort in reviewing our manuscript and for highlighting its importance. We have addressed the comments provided and revised the manuscript accordingly.

Reviewer 1

Overall: This paper addresses a critical issue of shared sanitation and its role in reaching the goal of safely managed sanitation by 2030. Understanding the extent of shared sanitation use in Ethiopia may support strategies for realising SDG6 and for understanding what the barriers to achieving this might be.

Response: Certainly, Thank you for your critical feedback and for recognizing the importance of this manuscript. We have addressed the comments provided and revised the manuscript accordingly.

Comment #1. I think that there is a useful paper in here, but there needs to be a bit more clarity about what is arising from the study, what is new in the study, and what is a confirmation of other studies on the issue of shared sanitation. It also needs to be clearer when the other literature is arising from a case study in a particular country, and so may not be generalisable (will clarify below in the line by line review when this comes up). I think that there are times that other studies are used to illustrate something that does NOT arise out of this study (for example on domestic violence) and this is confusing to the reader.

Response: Thank you for noting this. This study focused only on improved shared sanitation and its spatial distribution in Ethiopia. Providing shared sanitation services where improved sanitation services are limited could be a crucial first step in achieving the universal sanitation coverage aim outlined in the Sustainable Development Goals. We use only variables which have been associated with the previous studies on sanitation services.

Comment #2. The study could benefit from more clarity on the definitions used, particularly given the move from MDGs to SDGs and the greater focus on whether a service is safely managed rather than on the technologies used.

Response: Thank you for noting this. The definition of the outcome variable is clearly stated in the "Study Variables" section. We have revised it based on the DHS recode manual to read: "If a household uses any of the listed improved sanitation facilities and shares them with other households it is considered as improved shared sanitation services" (see page 7, lines 153-155).

Comment #3. One important clarification would be whether this study assumes that people who are not using SS are practising OD – is that correct? If so, that needs to be spelled out –

that SS is the better option. Because at the moment we just read, those who use SS and those who do not, but without the clarity of what the options are of those who do not use SS. This could be my lack of statistical knowledge, but even so, it would be good to clarify in the text. To the same end, it would be useful to have a graphic of the overall breakdown of sanitation used in Ethiopia – from SMS, to SS, to OD.

Response: Thank you for noting this. We have corrected it; accordingly, the following sentences clearly shows the outcome and controls. The outcome of this study is improved shared sanitation facilities whereas the control group are all unimproved sanitation facilities which includes OD, and all unimproved sanitation services such as Pit latrines without slab/open pits, Bucket toilets, hanging toilet/latrines and others. We modified and revised the title and all sections of the manuscript accordingly. "***Spatial distribution and determinants of improved shared sanitation facilities among households in Ethiopia: Using 2019 mini-Ethiopian Demographic and Health Survey***"

The study population consisted of all houses that had unimproved sanitation services evaluated during the 2019 mini-EDHS survey (7,561). This includes, shared improved sanitation services (1,276), unimproved sanitation facilities (3,442), and open defecation (OD) (2,843). From the total 8,663, households included in the 2019 mini-EDHS survey, 1,102 households did have improved but not shared sanitation facilities and were excluded from the analysis. Ultimately, a sample of 7,561 (weighted 7,770) households was included in the analysis. (see Page 6 line no 146-151).

Comment #4. For the results/discussion/conclusion, you could provide recommendations that arise from this study – what does the data tell you that will help planners etc. to increase access to shared sanitation, or more specifically to move people from OD to shared sanitation?

Response: Thank you for noting this. We have added a recommendations section and incorporated the issues that need to be addressed by the concerned bodies and stakeholders. Revised according to the following "*The government of Ethiopia and other stakeholders should provide economic support to low-income households to construct shared sanitation facilities. Additionally, policy should emphasize user awareness and practices through education to enhance access to and usage of improved shared sanitation facilities, particularly in rural residences and large central regions*. Further qualitative studies are needed to explore the behavioural and socio-cultural factors that may prevent individuals from utilizing improved shared sanitation facilities" (see page 20, lines 385-391).

Abstract

Comment #5. Line 29 -33 This starts as a good argument for including shared toilets in statistics but lines 33-34 do not clarify how understanding the prevalence of shared sanitation (SS) further supports this argument.

Response: Thank you for highlighting this important point. We have addressed the identified gap and incorporated it to support the argument. Corrected as follows: "Evidences are limited on the distribution of shared sanitation services and its determinants in Ethiopia. Therefore, this study aimed to assess the magnitude of shared toilet facilities and their determinants among households in Ethiopia.*"* (see Abstract page 2, lines 30-31 And page 5 line number line number 118-119 in the introduction section).

Comment #6. Lines 35-50 – I am not a statistician so cannot comment

Response: Thank you for your honest feedback, we double-checked it.

Comment #7. Lines 51-54, It would be useful to have a few headline results – what age or education means for the prevalence of shared sanitation

Response: Thank you for your valuable comment. We have corrected it as follows: "Being 55 years or older, having secondary or higher education, having middle or rich wealth status, living in urban areas, and residing in metropolitan or peripheral regions were significantly associated with the usage of shared toilet facilities.*" (see page 3, lines 51-53).*

Introduction

Comment #8.

I recommend that the discussion on the move from MDGs to SDGs happens here, and what that means for the indicators etc., as the data is from the SDG era.

e.g. lines 70-78 – are these MDG definitions, or SDG definitions

Response: Thank you for your response The SDG definition states that "*The Sustainable Development Goals target 6.2 calls for universal access to sanitation by 2030 and no child should die or get sick as a result of drinking contaminated drinking water, and/or being exposed to other people's excreta"*

Comment #9. Line 79 – check the numbers, something is not right – an increase of 3-6 million is not worth mentioning if the total using SS is 600 million. Line 80-82 – check the numbers 0.96 is not 18% of the population of SSA

Response: Revised accordingly as "Whereas in Sub-Saharan African countries households who used shared toilet facilities climbed from 0.64 million to 0.96 million, which was 0.08% of all households" *(see page 2, lines 93-94).*

Comment #10. Line86 – is a word missing after 'easy'

Response: We thank you. It was a grammatical and typing error. Modification has been made throughout the document.

Comment #11. Lines 103-104 – what is the source of this statement (it is not SDG 6.2 as suggested by the reference)

Response: We thank you and revised the source.

https://iris.who.int/bitstream/handle/10665/258617/9789241512893-eng.pdf?sequence=1

Comment #12: Line 110 – they may be superior to OD – but what are the criteria?

Response: Thank you for raising this point: improved shared sanitation facilities are superior to open defecation for several reasons:

- They reduce the incidence of open defecation, lowering exposure to harmful pathogens and disease spread.
- They offer more privacy and dignity than open defecation, which occurs in public spaces.
- They generally cause less environmental pollution compared to open defecation, which contaminates soil and water sources.
- They provide a more structured and accessible option, especially in urban or densely populated areas.

Comment #12: Lines 115-117 – are you drawing a link between using, or not using SS and child mortality? Are you drawing that from your data?

Response: Thank you for your interesting question, it is not from our data. It is based on the reference since it is an introduction, we want to show its burden. However, we revised the child mortality section.

Comment #13: Lines 118-119 – what is the relevance for planners – so that they can target particular households?

Response: Thank you for your interesting question. Knowing the magnitude of improved shared toilet facilities and their contributing factors in Ethiopia helps program planners allocate resources more effectively, design targeted interventions, and develop relevant policies. This understanding enables better planning and decision-making, leading to improved sanitation outcomes, enhanced public health, and more efficient use of resources by addressing the specific needs and challenges faced by different communities *(see page 5, lines 121-123).*

Comment #14: Lines 138-143 – I am confused by these numbers – there are 8663 households across 645 EAs? That doesn't make 20-30 households per EA – please clarify here.

Response: Thank you very much for showing the mistake we made; Now we revised accordingly, as the following "*In the first stage, a total of 305 EAs (93 in urban areas and 212 in rural areas) were selected with probability proportional to EA size. The second stage was the careful selection of, on average, 25–30 homes (based on the 2019 EPHC frame)" (see page 6, lines 141-143).Though* the revised sample size is 7,561, in the 2019 mini-EDHS 8663 households were included. Then 8,863/305=29 households on average.

Comment #15. I regret that the rest of the methods section doesn't mean anything to me!

Response: No worries; thank you very much for focusing on your experts.

Results

Comment #16. Line 212 – can you define what a male head of household denotes and what a female head of household denotes? Is a head of household only female if there is no adult male in the house? Or how is it defined?

Response: Thank you for seeking clarification. Yes, as you mentioned, the sociodemographic variables of the household data/record (HR) in DHS are collected for the head of households only. Then in the Ethiopian context, mostly if both male (father) and female (mother) are present in the household, the male is considered the household head. However, if the female is leading the household alone, she is recognized as the household head. *(See Table 2)*

Comment #17: Line 215 I think here you could summarise up front that an increase in age, and decrease in education, wealth etc. leads to a household being more likely to use SS. Then you can go into the detail

Response: Thank you for this important comment. Revised in the manuscript as the following: "Based on the results from Model III, there was a positive association between improved shared sanitation facilities and several factors such as individuals with higher educational attainment and from greater household wealth, those living in urban areas and metropolitan regions". *(see page 13, lines 244-246).*

Comment #18: Lines 235 – 239 Where is the risk of DV data coming from? And is SS a DV risk or a GBV risk? What is the correlation? does this suggest a correlation? A causation? Or just two random connected indicators of increased vulnerability?

Response: Thank you for your comments, and we apologize for any confusion. We are not entirely sure about the abbreviations "DV" and "GBV" you used. However, if "DV" refers to

the dependent variable, it implies that improved shared sanitation facilities in our context. The risk associated with the dependent variable is influenced by the cluster in which individuals reside. Since we used Enumeration Area (EA) or cluster as a random variable, the variations observed in the null model (e.g., ICC, MOR) were not due to random chance but rather to the variability of the random variable, which was the cluster or enumeration area. This indicates there was a correlation or association between the random variable (cluster) and the dependent variable. This is accounted for by our multilevel analysis.

Comment #19: Line 244 – 247 – write this as clearly as possible also for a non-statistician audience – as I understand it, this is your main finding.

Response: Thank you for your suggestion: Modifications and clarification have been made in the manuscript as follows: "Based on the results from Model III, there was a positive association between improved shared sanitation facilities and several factors such as individuals with higher educational attainment and from greater household wealth, those living in urban areas and metropolitan regions". *(see page 13, lines 244-246).*

Comment #20: From line 306 onwards I cannot comment! Sorry!

Response: No worries; thank you very much for focusing on your experts.

Discussion

Comment #21: Again – I think it needs to be clarified what the non-SS households are using, to be clear that it is worse than SS.

Response: Thank you for your suggestion: We have corrected it; accordingly, the following sentences show the outcome and controls. The outcome of this study is improved shared sanitation facilities whereas the control group are all unimproved sanitation facilities which includes OD, and all unimproved sanitation services such as Pit latrines without slab/open pits, Bucket toilets, hanging toilet/latrines and others. (see table 1).

Comment #22: Line 329 - this is not a strong reference, as it is a webpage without links to the claims it references.

Response: Thank you for your suggestion: revised it accordingly.

Comment #23: Lines 331-332 Did you get this from the data in the study? Or from somewhere else (if so reference) Also add "Studies in Zambia and Ethiopia show….." I think

that the context is critical.

Response: We have got it from previous studies, and we cited references accordingly as the following. *"*Moreover, a higher level of education enhances awareness and fosters positive attitudes towards choosing relatively more upgraded sanitation facilities [36]. Specifically, educated women are more likely to prefer safe and sanitary facilities that offer privacy and maintain good quality during their menstrual cycle [37]. Moreover, well-educated households with higher incomes had better access to upgraded sanitation facilities [38]. *"* (see page 18, lines 330-335).

Comment #24: Line 334 – is this an assumption? |Or does education lead to households using SS (which is what your data says, I think).

Response: Thank you for your valuable comments. Revised accordingly and removed the OD from the revised manuscript. Our data says household head education can lead to the use of Improved shared sanitation services and it is already expressed in the same paragraph.

Comment #25: Line 341 – the reference you use for the tragedy of the commons is rather old-fashioned, and the tragedy of the commons itself may not be relevant to sanitation usage. More work has been done on this more recently, e.g. by Ostrom, or by McGranahan.

Line 344 – 346 – is this speculation or from the data?

Line 346-348 it would be better to say "A study in Kenya shows…." Because otherwise it can be taken as a general truth and it may not be.

Response: Thank you for your valuable comments. We've updated and corrected the manuscript following the re-analysis. The number of family members in the household was found to be non-significant, and this discussion has been removed from the manuscript.

Comment #26: Line 347 – is 'consistent income' one of the indicators in the data, it's the first time I see the qualifier 'consistent'?

Response: Thank you for your comments. We discuss this issue for the association of rural residence and low improved sanitation facility usage from previous studies as the following "*On the other side, sanitation is an investment and peri-urban inhabitants made the effort to get improved toilet facilities because they had relatively consistent income to support their planning."* (see page 18, lines 352-354).

Comment #27: Lines 358 – 361 – this seems to be a circular argument – rewrite for clarity.

Response: Thank you for the comment. The information has been modified accordingly.

Comment #28: Line 361 – does 'governments' here refer to local governments in Ethiopia or other national governments?

Response: Both Ethiopian and other national governments do not prioritize rural sanitation specifically in their national agenda.

Comment #29: Line 365 – blanket statement not supported by the reference as far as I can see

Response: Thank you. Just reference is cited.

Conclusion

Comment #30: The conclusion could usefully contain some recommendations arising from the study – how can planners/government used this data analysis to make changes to how they promote SS? What are the lessons that can be acted upon?

Response: Thank you for your comments; we revised the recommendation in the revised manuscript as the following "The government of Ethiopia and other stakeholders should provide economic support to low-income households and rural residents to construct improved shared sanitation facilities. Additionally, policy should emphasize user awareness and practices through financial and education literacy to enhance access to and usage of improved shared sanitation facilities, particularly in rural residences and large central regions. Further qualitative studies are needed to explore the behavioural and socio-cultural factors that may prevent individuals from utilizing improved shared sanitation facilities (see page 20, lines 385-391).

Comment #31: What still remains to be researched and analysed. What is new from this study, and what confirms previous studies?

Response: Thank you for the interesting question. We reviewed various pieces of literature on the concept of shared sanitation and included them in the introduction. However, we found that no studies have specifically addressed the distribution of shared toilet facilities and their determinant factors in Ethiopia. Therefore, this study aims to address this gap. The new findings of this study are: we explored the spatial distribution and identified the determinants of improved shared sanitation facilities among households in Ethiopia. We suggested that further qualitative studies are needed to explore the behavioural and socio-cultural factors that may prevent individuals from utilizing improved shared sanitation facilities. (See page 20, lines 390-391).

Comment #33: Also – be careful what assumptions are embedded in the analysis – for

example, if women care so much about sanitation, why do households with a male head of household have increased access to SS?

Response: Thank you for the comment. However, in our analysis, the gender of head of the household has no association with improved shared sanitation facility usage.

Comment #22: Another still to be resolved question, critical for acceptance of SS as safely managed sanitation is what types of shared toilet facility people are using, and whether they are properly maintained? I believe that the main issue with shared sanitation is that it is difficult to judge if they are indeed improved, or safely managed or barely better than OD.

Response: Thank you for the comment: The analysis included improved shared toilet facilities as an outcome variable. We revised accordingly starting from the title of the manuscript. *"Spatial distribution and determinants of improved shared sanitation facilities among households in Ethiopia: Using 2019 mini-Ethiopian Demographic and Health Survey"*

Reviewer #2:

Comment #1: Summary and overall impression.

The paper addresses important issue of shared sanitation facilities and puts it in the individual household and community perspective. As it uses well recognised and reliable EDHS survey data, the analysis should yield dependable results that could support policy decision makers and other stakeholders when developing interventions at local and country level. It should also provide reference points for researchers and sanitation experts alike.

From a reader point of view there is a confusion related to the definition of shared sanitation and improved sanitation. I find it difficult to see how shared sanitation is assessed in the context of the type of primary toilet type available to the household. In terms of proportions, methodology and modelling sections are broad and detailed but the outcome of the analysis that is supposed to address research questions is incomplete.

Although the discussion section provides many valid points, the paper in my opinion requires major revisions to communicate its points clearly and develop more substantial recommendations that should be developed in the closing (conclusions) section.

Grammar, spelling mistakes and clarity of writing style must be addressed as well. Evidence and examples

Response: Thank you for recognizing the importance of this manuscript and finally your overall critical feedback.

- The definition of improved shared sanitation is critically revised in the revised manuscript Table 1. The outcome of this study is improved but shared sanitation facilities. If a household uses any of the listed -improved sanitation facilities but shares

them with other households, it is considered as improved shared sanitation facilities. The control group are all unimproved sanitation facilities which include OD, and sanitation services such as Pit latrines without slab/open pits, Bucket toilets, hanging toilets/latrines and others. We revised accordingly starting from the title of the manuscript

- Discussion and conclusion: The conclusion of the manuscript is revised based on the research questions and or objects. *We have 3 research questions. 1. What was the magnitude of improved shared sanitation facilities in Ethiopia? 2. What do the spatial distributions of improved shared sanitation facilities in Ethiopia look like? 3. What are the associated factors with improved shared sanitation facilities?*
- Therefore, we revised all the results and discussions accordingly as the following *"The prevalence of improved shared sanitation services usage among households in Ethiopia is relatively low. Age, educational attainment of the household head, wealth status, residence, and region were found to be significantly associated factors with improved shared sanitation facilities in Ethiopia. Moreover, there was a non-random distribution of improved shared sanitation services in Ethiopia mainly found in Addis Ababa, Dirie Dawa".* (see page 20, lines 381-385).
- Grammar and spelling mistakes: We have revised all the sections of the manuscript for grammar and spelling errors.

Major issues

Comment #2: The introduction that is to include an overview of previous research is narrow and lacks currency. It should have a clearer structure and include more recent research-based evidence. Indeed, the most recent reference cited is from 2022 (only one position).

I recommend Shared sanitation in informal settlements: A systematic review and meta-analysis of prevalence, preferences, and quality. https://doi.org/10.1016/j.ijheh.2024.114392. to provide broader perspective for this paper in the context of previously published research.

Response: Thank you for your valuable feedback and for providing the important reference article. We have incorporated and cited this article in the revised manuscript and have updated the introduction to reflect the latest literatures (See page 4 line No 85-88 and page 17 line 310 in discussion part).

Comment #3: Definition of shared sanitation is unclear – contradicting statements 78 vs 75.

Response: Thank you for your critical comments. The definition of improved shared sanitation is critically revised in the revised manuscript Table 1. The outcome of this study is improved but shared sanitation facilities. If a household uses any of the listed -improved sanitation facilities but shares them with other households (literally unimproved), it is considered as improved shared sanitation facilities.(Improved sanitation service, if shared=unimproved).For more evidence (see the introduction part page 5 line no 82-84).The control group are all other unimproved sanitation facilities which include OD, and sanitation services such as Pit latrines without slab/open pits, Bucket toilets, hanging toilets/latrines and others. We revised accordingly starting from the title of the manuscript.

Statements 78 vs 75 are not contradicted. If improved sanitation facilities are shared between two or more households, it is considered unimproved. Therefore, the term "improved shared sanitation" is one of unimproved sanitation services. The difference from other unimproved services such as OD is, that it is the acceptable and recommended service for refugees, slum areas and people with low socioeconomic status.

Comment #4: Research question 2 (118, 119) is unclear – contributing factors (contributing to what?).

Response: Thank you for your critical comments. The study is a cross-sectional study, we can assess only the association between Dependent Variable and independent variables. Therefore, we revised it accordingly as the following "What factors are associated with improved shared sanitation facilities?"

Comment #5:

The results section needs to be re-written to address gaps (e.g. explanation of how cluster with high risk of domestic violence was identified/taken from original data set?) and improve readability by providing more detailed interpretations (see e.g. 257, 258).

Response: Thank you for your comments. However, we had no domestic violence factor or outcome variables. Apologise I couldn't get your comments. For any variable generation from the original data set you can refer table **Table 2: a list of the study's variables along with an explanation of each measurement**

Minor issues

Comment #6: More recent population figures are available for Ethiopia now; 2023 as opposed to cited value for 2021 (131).

Response: Thank you for your comments: Revised based on the 2024, worldometere report. With 1.1 million square kilometres under its belt and an expected 132,059,767 inhabitants in 2024, Ethiopia is the second most populated country in Africa next to Nigeria

Comment #5: Table 1 (149) does not present the logic of questions well.

Response: Thank you for your comments, we revised Table 1 as the following:

Table 1: Questions to measure improved but shared/ limited sanitation service.

Outcome variable (Shared/ limited sanitation service)	***Control group/comparators***
Did the toilet have flush/pour flush to the piped sewer system?	✓ *Unimproved sanitation services such as*<br>○ *Pit latrine without slab/open pit*<br>○ *Bucket toilet*<br>○ *Hanging toilet/latrine*<br>○ *Other*<br>✓ *Open defecation: no facility/bush/field*
Did the toilet have flush/pour flush to the septic tank?	
Did the toilet have a flush to somewhere else?	
Did the toilet have flush/pour flush to the pit latrine?	
Did the toilet have a flush, don't know where?	
Was the toilet Ventilated Improved Pit (VIP) latrine?	
Was the toilet pit latrine with a slab?	
Was the toilet composting toilet?	
If a household uses any of the listed above-improved sanitation facilities but shares them with other households.	

Miscellaneous comments

Comment #5: Formatting of references requires attention – there are issues with the numbers and completeness of the records.

Response: Thank you so much, we revised the reference format and numbering accordingly.
