# Supplementary material for: Spatial distribution and determinants of improved shared sanitation facilities among households in Ethiopia: Using 2019 mini-Ethiopian Demographic and Health Survey
Source: PLoS One. 2025 Jan 13;20(1):e0315860. doi: 10.1371/journal.pone.0315860 (PMC11730421; doi:10.1371/journal.pone.0315860)
Supplement: Attachment — Submitted filename: Response to Reviewers.docx [file pone.0315860.s003.docx]

Date: December 14, 2024

Alison Parker

Academic Editor of Plos One Journal

**Re:** ***Spatial distribution and determinants of improved shared toilet facilities among households in Ethiopia: Using 2019 mini-Ethiopian Demographic and Health Survey (Submission ID:* PONE-D-24-20290*)***

Dear Editor,

We are grateful for the opportunity to revise our manuscript for further consideration for publication in Plos One Journal.

We have addressed the reviewer's comments and suggestions. Our point-by-point response describes all changes in the manuscript text. We have indicated the changes in track changes in the revised manuscript. We hope that you will find the revised manuscript acceptable for publication.

Yours sincerely,

Baye Tsegaye Amlak

Corresponding Author

**A point-by-point response to the reviewer’s comment**

**Reviewer #3:**

Most of the comments were addressed correctly. Here are additional comments with regards to revision1:

Thank you for your feedback and for recognizing the importance of this manuscript. We have addressed the comments provided and revised the manuscript accordingly.
**Comment #1.** In the response to reviewers' comments, you gave 3 research questions: " 1. What was the magnitude of improved shared sanitation facilities in Ethiopia? 2. What do the spatial distributions of improved shared sanitation facilities in Ethiopia look like? 3. What are
the associated factors with improved shared sanitation facilities?" In the text you have only 2 questions 119-121.

Response: Thank you for noting this. In the revised manuscript, we have included three research questions? 1. To determine magnitude, 2. Spatial distribution, 3. determinants or factors. We have revised it as a short description as the following. “*This study aims to address the following research questions: What is the* ***magnitude*** *and* ***spatial distribution*** *of improved shared sanitation facilities in Ethiopia? What* ***factors*** *are associated with improved shared sanitation facilities? See page 5 line number 119.* Furthermore, those research questions were clearly answered and indicated in the manuscript (Magnitude of improved shared sanitation(Page 12 line number 239), factors associated with it(page 15 table 4), spatial distribution page 16 line number 302).

**Comment #2.** In your Multilevel model results you state: "A household from a cluster with a high risk of domestic violence had 28.75 times higher odds of having shared toilet facilities than a household from a cluster with a lower risk of shared toilet facilities, according to the MOR value in the null model (28.75), on the other hand, if you randomly selected two households from two different clusters. "You can see you are referring to a cluster with high risk of domestic violence. How these cluster characteristics has been arrived to? Is it one of the characteristics of EA? This needs to be clarified before you report your results.

**Response:** We thank the reviewer. “*Domestic violence*” was the wrong word incorporated in the document. We have revised the document accordingly. Here is the comment. *“According to the MOR value in the null model (28.75), a household from a high-risk cluster had 28.75 times higher odds of having shared toilet facilities compared to a household from a low-risk cluster”. Please, see* line numbers 238-240.

**Comment #3.** There is a need for further proofreading of the text. See e.g. the paragraph copied above for style and clarity and 241 (valve - should be value?).

Response: Thank you for noting this. We have corrected the specified spelling. Moreover, we have proofread all sections of the documents.

**Comment #4.** Referencing - also requires additional check to make sure all statements made are linked to this study results or specific source(s) see e.g. 322.

Response: Thank you for your comment. Even though the EDHS report reported the magnitude of open defecation, we have revised and changed it with the updated articles.

*“Spatiotemporal distribution and determinants of open defecation among households in Ethiopia: A Mixed effect and spatial analysis”*

https://journals.plos.org/plosone/article?id=10.1371/journal.pone.0268342

**Comment #5.** Recommendations are basic and could have been developed better taking into account findings and discussion. (E.g. if other countries mentioned have higher standards of sanitation are there any lessons to be learned from their experiences?

Response: Thank you for your comment.

We have addressed and revised based on your comments
